# Tumor-associated osteoclasts converge on a matrix-sulfation program across human bone tumors

**DOI:** 10.3389/fcell.2026.1914698

**Published:** 2026-09-10

**Authors:** Mengyuan Lei, Shuya Chang, Ningjing Lei, Zhenhua Zhang, Chenghan Luo

**Affiliations:** 1 Department of Health Management Medicine, The First Affiliated Hospital of Zhengzhou University, Zhengzhou, Henan, China; 2 The First Clinical Medical College of Zhengzhou University, Zhengzhou, China; 3 Department of Microbiology and Immunology, School of Basic Medical Sciences, Zhengzhou University, Zhengzhou, Henan, China; 4 Department of Computational Biology and Medical Science, Graduate School of Frontier Sciences, The University of Tokyo, Tokyo, Japan; 5 Department of Orthopedics, The First Affiliated Hospital of Zhengzhou University, Zhengzhou, Henan, China

**Keywords:** bone metastasis, glycosaminoglycan sulfation, human bone tumors, osteoclastogenesis, osteoclasts, single-cell RNA sequencing

## Abstract

**Introduction:**

Osteoclasts mediate the bone destruction that drives much of the morbidity of giant-cell tumor of bone, osteosarcoma, and skeletal metastasis, yet these large multinucleated cells are lost in droplet single-cell data and have never been defined as a disease cell state.

**Methods:**

We reanalyzed 378,936 cells from osteoclast-rich human bone tumors and reference datasets, using within-tumor comparisons and independent validation analyses to characterize shared osteoclast transcriptional programs across the three tumor classes.

**Results:**

Tumor osteoclasts from all three tumor classes converged on a shared 74-gene transcriptional program. The program showed high scores in osteoclasts and low scores in neighboring myeloid cells, reproduced across four cancers of origin and in an independent cohort of 38,361 osteoclasts, and centered on a glycosaminoglycan-sulfation and mineralization module comprising PAPSS2, UST, EXT1, and FAM20C. The enrichment held across donors, the program increased as human monocytes differentiated into osteoclasts, and it tracked mature-osteoclast abundance in an independent spatial cohort of human osteosarcoma. Osteoclasts expressing the program were associated with a collagen and CD44 signaling neighborhood, retained the canonical regulators PU.1, MITF, and NFATc1, and occupied the terminus of osteoclastogenesis.

**Discussion:**

Across biologically distinct bone cancers, tumor osteoclasts share a reproducible, maturation-associated state centered on matrix-sulfation biology.

## Introduction

Osteoclasts are the large, multinucleated cells of the monocyte–macrophage lineage that resorb bone. Their differentiation runs through a tightly regulated RANKL and M-CSF program acting on NFATc1 and AP-1 (Takayanagi, 2007; Kawaida et al., 2003), and its dysregulation produces the osteolysis of osteoporosis, the erosions of rheumatoid arthritis, and the bone destruction behind much of the clinical burden of skeletal tumors. The core regulators of osteoclastogenesis are well known, yet the osteoclast remains one of the least accessible cells in human disease tissue. Large, strongly adherent, and multinucleated osteoclasts are preferentially destroyed during the tissue dissociation that feeds droplet single-cell assays, so they are scarce or absent from the very atlases now used to define cell states (McDonald et al., 2021).

Single-cell studies have begun to characterize the osteoclast and myeloid landscapes of bone tumors. Bone-metastasis atlases describe macrophage and osteoclast ecosystems, including MMP19-positive, RANK-positive tumor-associated macrophages, while studies on giant-cell tumor of bone (GCTB) and osteosarcoma have profiled osteoclast-rich tumors, denosumab-associated recurrence, proliferative osteoclast subgroups, and therapy-resistant osteoclast precursors (Sun et al., 2024; Liu F. et al., 2025; 2026; Wu et al., 2025; Hu et al., 2026a; 2026b). This study shows that osteoclast and macrophage-like states are clinically relevant, but it leaves a basic question open. Many bone-resorption genes are shared among mature osteoclasts, monocytes, macrophages, and tumor-associated macrophages, and most analyses remain within a single disease. Whether biologically distinct bone tumors converge on a common mature-osteoclast state, and what that state is made of, has not been established.

One obstacle has kept this state out of reach. Tissue dissociated from a collagen-rich, immune-infiltrated tumor carries abundant ambient RNA, and a conventional tumor-versus-normal comparison cannot distinguish an osteoclast-intrinsic signal from this contamination. Trajectory inference, clustering, regulon analysis, and tumor-versus-normal differential expression each return an ordered answer, but none carries a control that separates real state from contamination. The problem is most pronounced for osteoclasts, which are rare and especially prone to ambient pickup, so the default analysis tends to report contamination as discovery, and tumor-osteoclast signatures have been correspondingly difficult to reproduce.

We reasoned that the contaminating signal is shared by all of the cells co-dissociated from one tumor and that comparing osteoclasts with the myeloid cells of their own tumor would cancel much of it. Pairing this within-tumor comparison with replication in held-out tumors, an all-compartment specificity test, donor-level inference, external differentiation, osteoblast and spatial references, and observational regulatory analysis, we re-analyzed an osteoclast-rich atlas spanning three bone-tumor classes. The naive comparison is dominated by an ambient artifact; in contrast, the within-tumor comparison recovers a focused 74-gene osteoclast program that is reproducible across cancers of origin in external cohorts and in intact human bone. We then resolve the program into functional modules, identify a glycosaminoglycan-sulfation and mineralization module as its osteoclast-enriched core, and place it within osteoclast maturation.

Across three classes of human bone tumor, mature osteoclasts share a reproducible 74-gene program that is not explained by myeloid identity or modeled ambient RNA, whose osteoclast-enriched core comprises a matrix-sulfation and mineralization module, and the program resides at the terminus of osteoclastogenesis alongside canonical osteoclast regulators and in association with the collagen-rich stroma.

## Results

### An osteoclast-rich atlas resolves mature osteoclasts in every bone-tumor context

**FIGURE 1:**
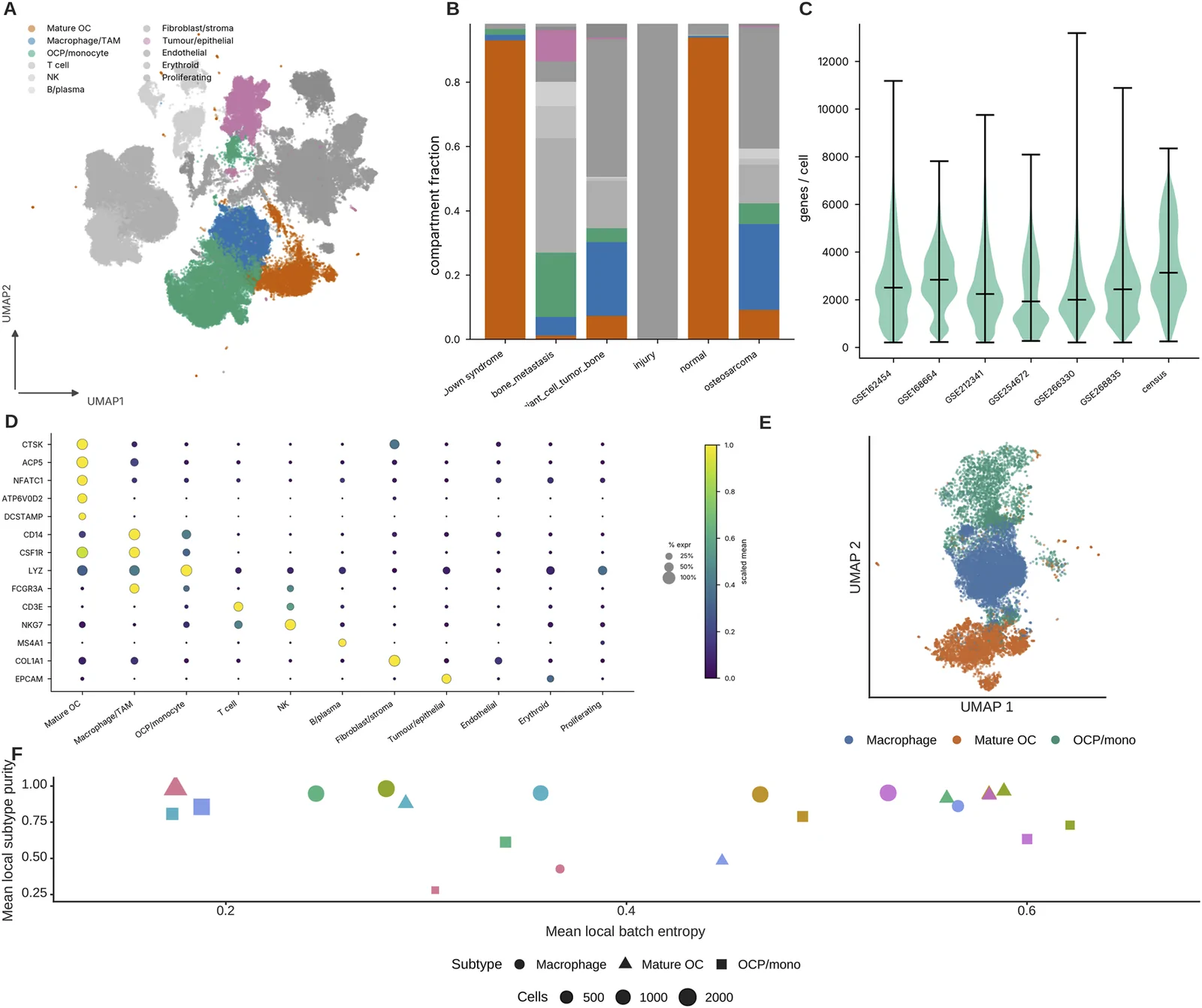
An osteoclast-rich cross-tumor atlas resolves mature osteoclasts in every tumor context. **(A)** Atlas UMAP colored by compartment (density contours overlaid); colors are used consistently throughout and are listed in Supplementary Table S1. **(B)** Compartment composition by tumor context (stacked proportions). **(C)** Detected genes per cell per cohort (violins, median). **(D)** Canonical-marker clustered dot plot (dot size, fraction expressing; color, per-gene scaled mean; columns ordered by hierarchical clustering). **(E)** Accession-balanced UMAP of the integrated representation, colored by osteoclast-related subtype (17,500 cells). **(F)** Local batch entropy against local subtype purity for the same cells; full metrics in Supplementary Figure S9 n = 378,936 cells; scVI integration, seed 0; sample silhouette −0.074.

We assembled 378,936 cells from GCTB, osteosarcoma, and osteolytic bone metastasis, together with osteoclast references, and integrated them with a variational model (Figure 1A; STAR methods). Giant-cell tumors are exceptionally osteoclast-rich (26.9% and 50.7% CTSK-positive and ACP5-positive cells, respectively, in the two reference cohorts), and the integrated atlas resolved 11 compartments: mature osteoclast, osteoclast-progenitor/monocyte, macrophage and tumor-associated macrophage, T cell, NK cell, B/plasma cell, fibroblast/stroma, endothelial, tumor/epithelial, erythroid, and proliferating cells; each compartment was defined using a canonical positive-marker panel and represented by a fixed color throughout the analysis, with the marker panels, annotation guards, cell counts, and display colors provided in Supplementary Table S1. The mature-osteoclast compartment comprised 19,852 cells and was present in every tumor context (Figures 1A,B), among which 17,548 entered the donor-level analysis set on which every donor-level statistic below is computed (6,322 GCTB; 4,308 reference; 3,461 osteosarcoma; 3,457 bone metastasis; Table 1). Mature osteoclasts exhibited the high transcript counts expected of multinucleated cells and expressed the fusion and resorption markers CTSK, ACP5, NFATC1, and ATP6V0D2, which remained restricted to this compartment under an annotation guard requiring the osteoclast signature to exceed stromal and osteoblast signatures (Figure 1D; STAR methods). Integration-mixed cohorts while preserving lineage structure: per-cohort gene-detection distributions were comparable (Figure 1C), the batch silhouette across samples was negative (−0.074), osteoclasts from different tumors co-embedded (Figure 1A), and the mature-osteoclast compartment showed the highest cross-cohort entropy of any compartment (1.76). An accession-balanced audit yielded a mean local subtype purity of 0.916 against an accession silhouette of 0.006 (Figures 1E,F; Supplementary Figure S9), and repeating the primary within-donor contrast seven times, omitting one accession each time, was positive in every run (Supplementary Figure S3). The atlas thus places biologically distinct bone tumors in one frame and holds enough mature osteoclasts to compare them.

**TABLE 1:** Cohorts and datasets. Mature OC (n) provides the six cohorts that comprise the atlas (GSE266330, GSE254672, GSE168664, GSE162454, GSE268835/GSE212341, and the CELLxGENE census reference), with mature osteoclasts entering the donor-level analysis set; these six entries sum to 17,548. GSE152048 and GSE143791 yield the osteoclasts scored in each external cohort. The remaining rows report the size of that resource in its own units.

Accession	Context	Tumor class	Platform	Role	Mature OC (n)	Access
GSE266330	Bone metastasis (+ adult control bone)	Carcinoma metastasis	Droplet scRNA-seq	Discovery + adult control	3,457 (including 134 adult control)	Open
GSE254672	Giant-cell tumor of bone	Non-malignant primary	Droplet scRNA-seq	Discovery	657	Open
GSE168664	Giant-cell tumor of bone	Non-malignant primary	Droplet scRNA-seq	Replication	735	Open
GSE162454	Osteosarcoma	Sarcoma	Droplet scRNA-seq	Replication (held-out disease)	3,461	Open
GSE268835/GSE212341	GCTB ± denosumab	Non-malignant primary	Droplet scRNA-seq	Denosumab-response control (anti-RANKL)	4,930 (3,643 + 1,287)	Open
GSE152048	Osteosarcoma	Sarcoma	Droplet scRNA-seq	External validation	38,361	Open
GSE143791	Prostate bone metastasis	Carcinoma metastasis	Droplet scRNA-seq	External (myeloid control)	262	Open
CELLxGENE census	Osteoclast reference (∼100% fetal)	Reference (sensitivity only)	Droplet scRNA-seq	Reference	4,308	Open
GSE246769	Human osteoclast differentiation (M-CSF/RANKL)	Non-tumor, *in vitro*	Bulk RNA-seq	External maturation series	8 donors, 29 libraries	Open
GSE253355	Adult bone-marrow niche	Non-tumor reference	Droplet scRNA-seq	External osteoblast-lineage reference	2,555 osteoblasts (12 donors)	Open
GSE293065	Osteosarcoma	Sarcoma	Visium spatial	External spatial corroboration	26 sections, 19 patients	Open
GSE284089	Normal femoral head	Non-tumor	Visium spatial (FFPE)	Normal-bone spatial reference	4,992 in-tissue spots	Open

### A within-tumor comparison uncovers a 74-gene osteoclast program behind a 1,703-gene contamination signature

**FIGURE 2:**
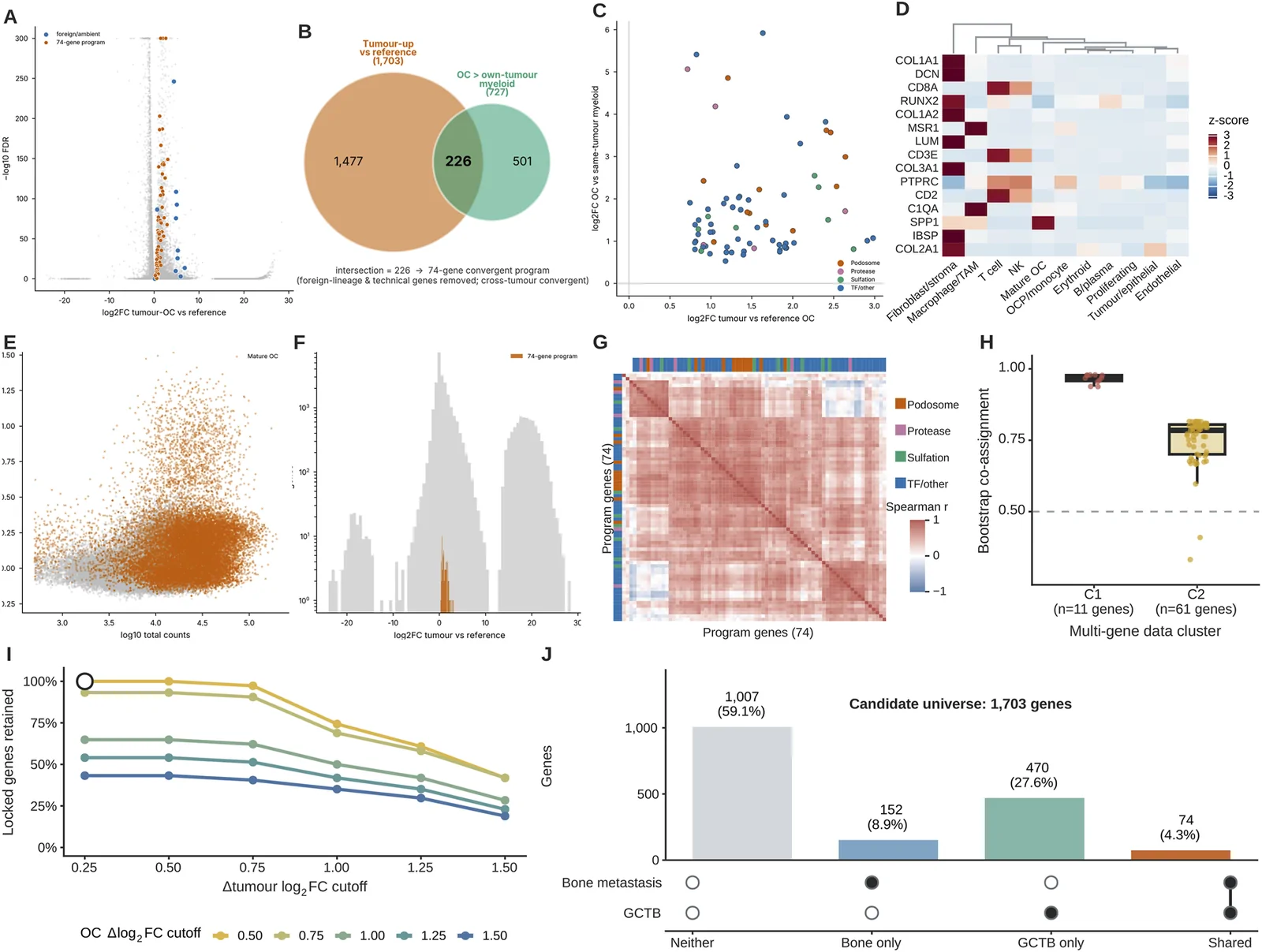
A within-tumor comparison uncovers a 74-gene osteoclast program behind a 1,703-gene contamination signature. **(A)** Volcano of tumor versus fetal-reference osteoclasts; foreign/ambient genes (blue) and the 74-gene program (orange). **(B)** The interaction filter as a set intersection (proportional Venn): tumor-up genes (1,703) ∩ osteoclast-over-myeloid genes (727) = 226, reduced to 74 after removing foreign-lineage and technical genes and requiring cross-tumor convergence. **(C)** Conjunction-criterion scatter; each of the 74 genes by its tumor-versus-reference and osteoclast-versus-myeloid log-fold change (both >0), colored by functional module. **(D)** Naive “hits” by compartment (clustered, row z-score); they peak in non-osteoclast compartments. **(E)** Program score versus read depth; osteoclast versus macrophage. **(F)** Distribution of tumor-versus-reference log-fold change for all genes versus the 74-gene program. **(G)** Donor-level correlation matrix of the 74 genes. **(H)** Bootstrap co-assignment stability of the two multi-gene clusters (72 of the 74 genes). **(I)** Retention of the 74 genes across stricter threshold combinations. **(J)** Partition of the 1,703-gene universe into the bone-metastasis branch (226), the giant-cell-tumor branch (544), and their cross-disease intersection (74). Details in Supplementary Figure S6. Two-sided Wilcoxon and Benjamini–Hochberg tests.

The obvious approach, comparing tumor osteoclasts with reference osteoclasts directly, returned 1,703 upregulated genes and near-perfect separation (AUROC 0.99; Figure 2A). The result is an illusion. Its leading genes are not osteoclast genes but markers of the surrounding tissue, including COL1A1, DCN, CD3E, RUNX2, and MSR1, all expressed more strongly in non-osteoclast compartments than in osteoclasts themselves (Figure 2D). The signature reflects ambient RNA from the collagen-rich, immune-infiltrated tumor, compounded by protocol differences between fresh tumor dissociation and the reference. A near-perfect, 1,703-gene separation is exactly what this contamination produces, and taking it at face value would report the tumor’s bulk tissue as osteoclast biology.

The contamination also points to its own remedy. If each measurement carries a tumor-wide ambient term shared by the cell types co-dissociated from one tumor (STAR methods, Equations 1, 2), then comparing tumor osteoclasts with reference osteoclasts confounds the genuine state with this ambient term; in contrast, comparing osteoclasts with myeloid cells from the same tumor shares the term and cancels much of its effect. We therefore required each program gene to be upregulated in tumor osteoclasts relative to reference osteoclasts and, at the same time, enriched in osteoclasts relative to their own tumor’s myeloid cells (STAR methods, Equation 3). The second comparison is a lineage contrast that substantially reduces shared ambient and foreign-lineage transcripts, leaving a set of genes that is firmly osteoclast-enriched; the residual ambient contribution is estimated directly with a formal generative model below.

Applying this requirement across the discovery tumors and removing foreign-lineage and technical genes collapsed the signature from 1,703 genes to 74 (Figures 2B,C). The two disease branches, filtered independently, returned 226 and 544 genes, and the 74 are their cross-disease intersection (Figure 2J); the excluded foreign-lineage and technical genes are listed with their exclusion rules in Supplementary Table S3. The 74 genes exhibit high tumor-versus-reference fold changes relative to all genes (Figure 2F), and their program score is not explained by sequencing depth (Figure 2E). The set is robust to the stringency of the filtering threshold, retaining 67 of the 74 genes when the thresholds were increased by 1.5-fold and 3-fold relative to those originally used, and its correlation structure is stable under donor-level bootstrapping (Figures 2G–I; Supplementary Figure S6). A single change in the comparator thus turns a tumor-wide contamination signature into a focused, osteoclast-enriched candidate program, validated and characterized by the following analyses.

### The osteoclast program is a coherent state of four functional modules

**FIGURE 3:**
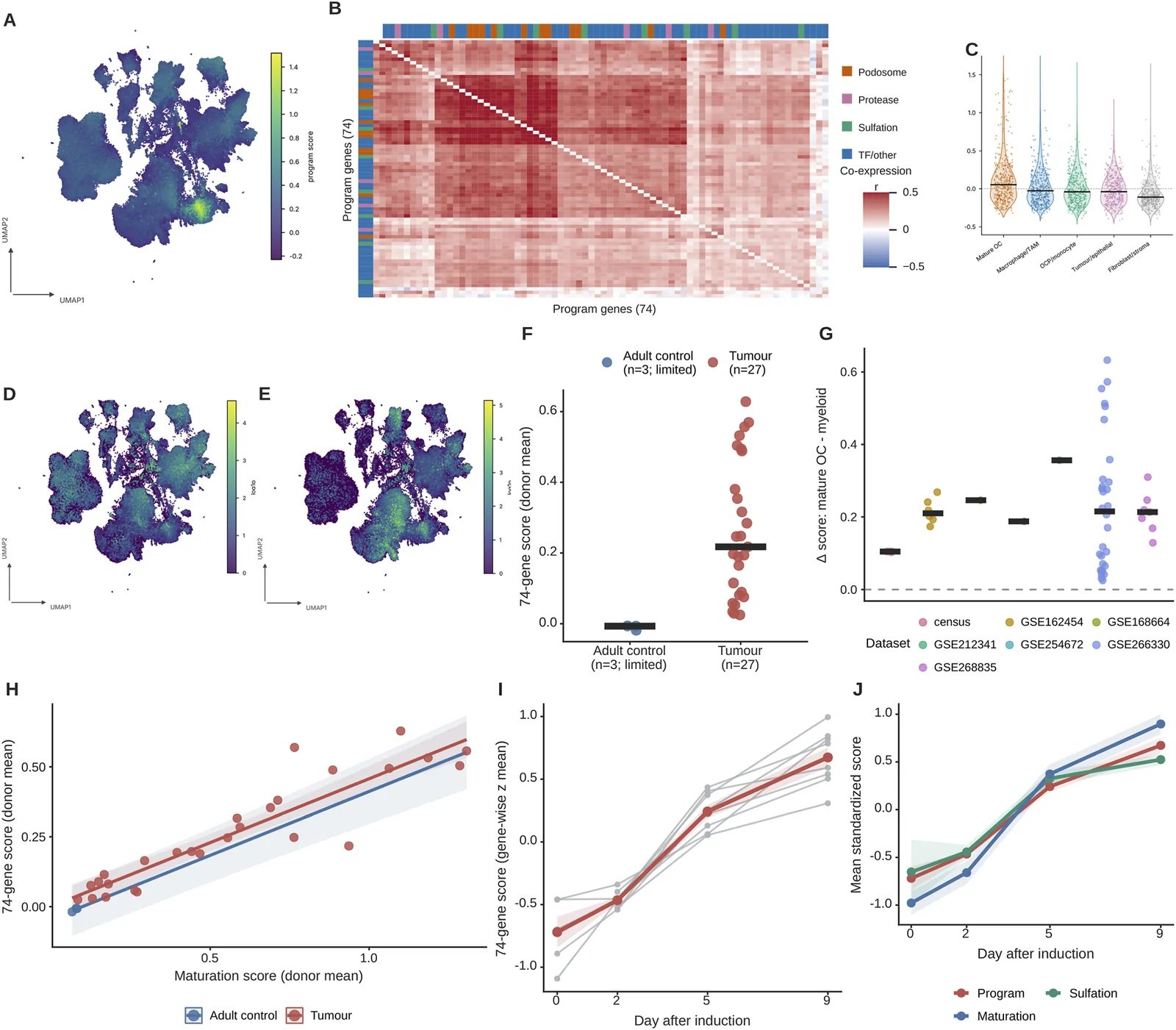
The osteoclast program is a coherent state of four functional modules. **(A)** Program score on the atlas UMAP (viridis). **(B)** Gene–gene co-expression of the 74 program genes within osteoclasts (Pearson correlation, hierarchically clustered; color bars, functional module), showing module block structure. **(C)** Sulfation-module score by compartment (sina: violin outline, points, median bar). **(D,E)** Expression of the podosome scaffold SH3PXD2A/TKS5 **(D)** and the sulfation enzyme PAPSS2 **(E)** on the UMAP. **(F)** Donor-level program score in tumor and adult-control donors (points, donors; bars, medians; Hedges’ g = 1.434). **(G)** Within-donor mature-osteoclast minus myeloid differences (50 donors, all positive; mean 0.219). **(H)** Donor-level model of the program score against maturation; with maturation held constant, the tumor coefficient was 0.045 (95% CI = −0.058 to 0.149; n = 30 donors). **(I)** Eight-donor time course of M-CSF- and RANKL-driven human osteoclast differentiation (GSE246769; days 0, 2, 5, and 9). **(J)** Program, sulfation-module, and canonical-maturation trajectories across the same time course (β = 0.163, 0.143, and 0.219 per day); gene-level results provided in Supplementary Figure S4.

The 74 genes behave as a coherent cell state, not a gene list (Figure 3). The program score was concentrated in mature osteoclasts as a graded gradient across the UMAP (Figure 3A), and within osteoclasts, the genes were co-expressed, partitioning by hierarchical clustering into four modules (Figure 3B): a podosome and actin-motility module (SH3PXD2A/TKS5, MYO1E, and DNM3), a secreted-protease module (MMP19), a glycosaminoglycan-sulfation and mineralization module (PAPSS2, UST, EXT1, and FAM20C), and a transcriptional module (JDP2, RUNX3, RFX8, and SOX4); the sulfation module was elevated in osteoclasts (Figure 3C). Set against the prior literature, the program combines confirmation with new ground: four genes are established osteoclast effectors, 18 have documented roles in bone or skeletal matrix biology, 12 are known myeloid or cancer genes, and for the remaining 40, our search returned no prior report linking them to osteoclasts (per-gene assignments and their basis in Supplementary Table S4; summary in Table 2). Those 40 were not the weaker members of the set: 30 of the 39 measurable genes in the differentiation series increased significantly by day 9, with a larger median induction than the 34 annotated genes (log2 fold change 1.83 against 1.05) and the same bootstrap co-assignment stability (0.80 against 0.79). The cytoskeletal module aligns directly with osteoclast biology since osteoclasts assemble podosomes and a sealing zone to resorb bone, and the invadosome scaffold TKS5 (SH3PXD2A) marks mature osteoclasts (Figure 3D) (Seals et al., 2005; Georgess et al., 2014; Feng et al., 2021). Several module genes have context-dependent roles in bone and myeloid cells, including MMP19 in RANK-positive macrophages, FAM20C in bone metastasis, and sulfate and glycosaminoglycan genes in mineralization (Zuo et al., 2021a; Lee et al., 2025; Hu et al., 2026a; Liu et al., 2026), so we treat each module as a component of the osteoclast state and assess its specificity in the compartment analyses that follow.

**TABLE 2:** Main results with confidence intervals.

Test	Metric	Value (95% CI)
Naive signature	up genes/AUROC	1,703/0.99 (ambient artefact)
Filtered program	genes	74
OC vs. macrophage compartment (selection axis; descriptive)	Cohen’s d	1.07 (1.06–1.09)
OC vs. own-tumor myeloid, depth-residualized (deconfounding axis)	Cohen’s d	2.60/2.13
MMP19, OC vs. macrophage	Cohen’s d	0.03 (not specific)
Sulfation module, OC vs. next-highest compartment	module-score d	0.36 (0.34–0.38)
Specific-core program (MMP19/ACTN2/COL27A1 removed), OC vs. macrophage	Cohen’s d	1.03 (1.01–1.05)
OC vs. macrophage, top-doublet-decile removed	Cohen’s d	1.08 (1.06–1.10)
External osteosarcoma (GSE152048)	Cohen’s d (OC vs. non-OC)	0.38 (0.36–0.39); 38,361 OC
External prostate bone-met (GSE143791)	Cohen’s d (OC vs. TAM)	2.74 (2.39–3.07); 262 OC
Held-out osteosarcoma vs. adult control	AUROC/Cohen’s d	0.83/1.16 (per-group range 0.83–0.91/0.95–1.56)
Maturation control (adult)	d_program/d_maturation/residualized	0.95/1.05/0.24
Gene re-recovery (held-out cohorts)	%	70–76
In silico KO (Δ; fold over own null)	NFATc1/JDP2/RFX8	−0.084 (8.0×) /−0.094 (3.1×) /−0.055 (8.2×)
Prior-literature class of 74 genes	OC-direct/bone-adjacent/myeloid/cancer/none	4/18/7/5/40
Donor-level OC vs. within-donor myeloid (primary unit)	mean paired difference	0.219 (0.180–0.263); 50/50 donors
Leave-one-dataset-out repetition of that contrast	mean paired difference	0.199–0.229; positive in 7/7 omissions
decontX-corrected program, OC vs. macrophage	donor median difference	0.137 (0.092–0.176); 58/72 donors
decontX-corrected sulfation module, OC vs. macrophage	donor median difference	0.203 (0.145–0.302); 58/72 donors
Human osteoclast differentiation (GSE246769)	program change per day	0.163 (0.143–0.184); 52/73 genes up at day 9
Sulfation module during differentiation	module change per day	0.143 (0.100–0.185)
Adult osteoblast reference (GSE253355)	osteoblast vs. Osteo-MSC, program	0.030 (0.012–0.047); 12 donors
Osteosarcoma spatial cohort (GSE293065)	patient median rho	0.074 (0.032–0.254); 17/19 patients
Spatial, annotation-independent 63 genes	patient median rho	0.051 (0.017–0.124); 16/19 patients
Normal-bone spatial (GSE284089)	sulfation vs. osteoclast score	0.174 (P = 2.0 × 10^−4^); adjusted 0.331
Tumor vs. adult control, donor level	Hedges' g	1.434 (1.155–1.883)
Threshold sensitivity of the 74 genes	retained at 1.5x/3x stricter cutoffs	67/74 genes
Regulator consensus (4 model classes, 2,000 nulls, and 7 omissions)	rank of RFX8	first on every consensus ranking

### The program is highest in osteoclasts and lowest in myeloid cells across eleven compartments

**FIGURE 4:**
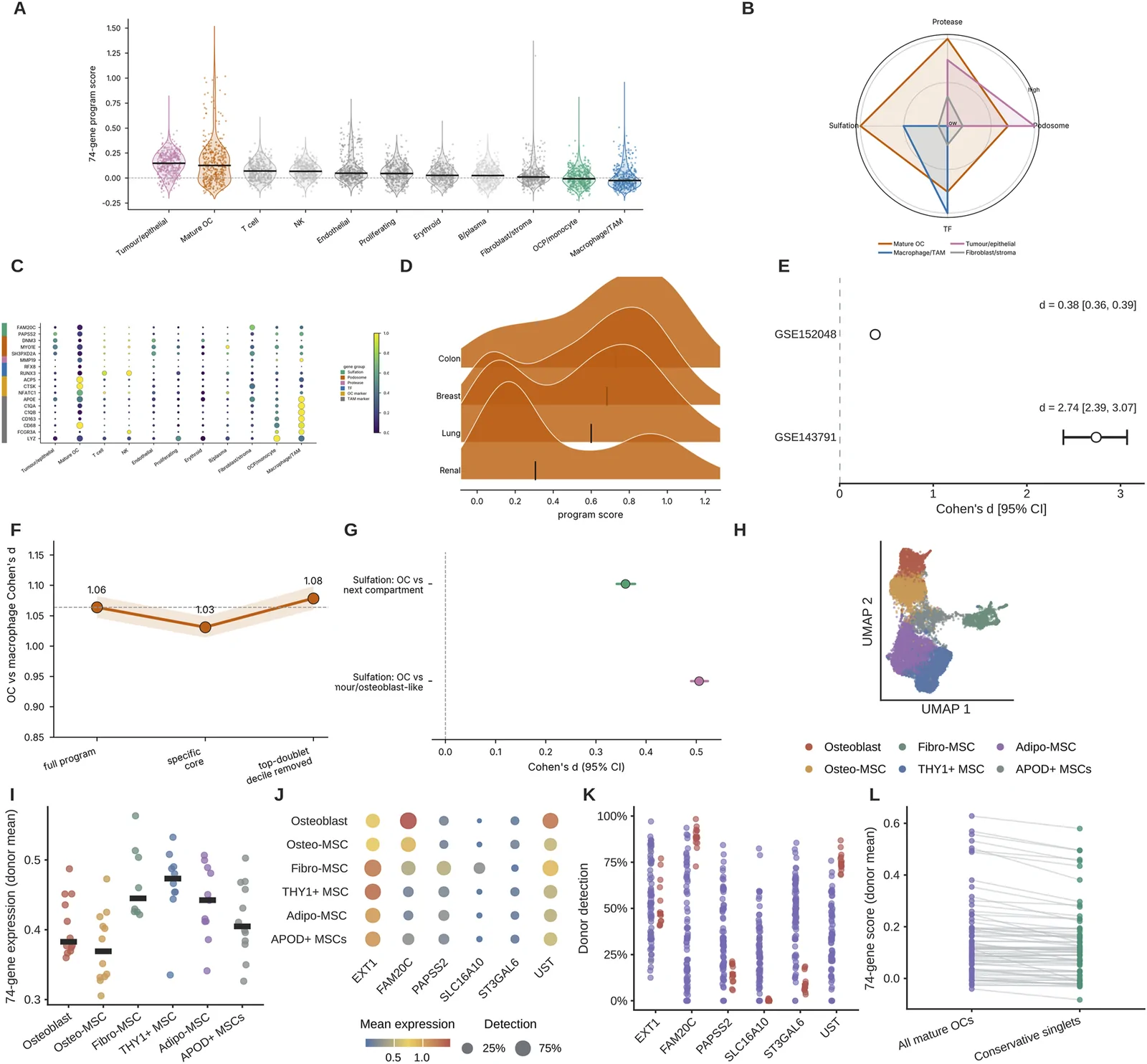
Across compartments, the program is osteoclast-high and myeloid-low; only its sulfation module is osteoclast-enriched; it reproduces across cancers and cohorts. **(A)** Program score across the 11 compartments (sina: violin, points, and median; orange, osteoclast; blue, macrophage/TAM; teal, OCP/monocyte; pink, tumor/epithelial; grey, remaining compartments); myeloid compartments score the lowest. **(B)** Functional-module fingerprint (radar) of osteoclasts versus the macrophage/TAM, tumor/epithelial, and stromal compartments (per-axis scaled); only the sulfation-mineralization axis is osteoclast-enriched, whereas the transcription-factor axis is highest in macrophages and the podosome axis in tumor/epithelial cells. **(C)** Program genes, MMP19, and macrophage markers across compartments (dot size, fraction expressing; color, per-gene scaled mean; left color strip, gene group). **(D)** Program-score distributions in osteoclasts by cancer of origin (ridgeline). **(E)** External replication effect sizes (forest; Cohen’s d, 95% bootstrap CI): osteosarcoma GSE152048 (38,361 osteoclasts) and prostate GSE143791 (osteoclast versus tumor-associated macrophage; n = 262 OC vs. 15,205 TAM). **(F)** Robustness of the osteoclast-versus-macrophage effect to gene and doublet controls (slopegraph; full program → specific-core → top-doublet-decile removed; band, 95% bootstrap CI). **(G)** Sulfation-module enrichment versus the next compartment and the osteoblast-like tumor/epithelial compartment (forest). **(H)** Published adult bone-marrow niche reference (GSE253355) colored by the authors’ own annotation. **(I)** Donor-level program and sulfation scores across published skeletal states in that reference (12 donors). **(J)** Candidate-gene expression and detection in the same reference. **(K)** Cross-study detection of the candidate genes in adult osteoblasts and atlas mature osteoclasts; values in Supplementary Figure S5. **(L)** Paired donor-level effect of the conservative osteoclast–osteoblast multiplet filter (72 donors; 83.3% of osteoclasts retained).

Because the program was defined in part against myeloid cells, we asked directly whether it is simply a myeloid signal. Scoring the 74 genes in every cell and comparing the eleven annotated compartments placed mature osteoclasts among the highest of all, while the macrophage, tumor-associated-macrophage, monocyte, and osteoclast-progenitor compartments, the sources of any ambient myeloid RNA, scored the lowest (osteoclast versus macrophage Cohen’s d = 1.07, 95% CI = 1.06–1.09; Figure 4A). Because cells from one donor are correlated, we repeated the contrast with the donor as the unit: mature osteoclasts exceeded the myeloid cells of their own donor in all 50 donors, with a mean paired difference of 0.219 (95% CI = 0.180–0.263; P = 7.8 × 10^−10^; Figure 3G; Supplementary Figure S3). The ordering argues against simple contamination in a second way: tumor and epithelial cells also exhibited an elevated score, which a myeloid-contamination model cannot produce, since under that model, the myeloid compartment would score the highest. Individual genes such as MMP19, TNFRSF11A/RANK, and CSF1R can mark macrophage or osteoclast states depending on context, so we leaned on the composite program score and on its separation from macrophage and TAM control signatures rather than on any single gene (Dai et al., 2025; Zhang et al., 2026a; 2026b).

### A sulfation and mineralization module is the osteoclast-enriched core of the program

Resolving the program module by module localized its osteoclast-enriched core (Figures 4B,C). Of the four modules, the glycosaminoglycan-sulfation and mineralization module was the one elevated in mature osteoclasts above every other compartment (module score 0.11 in osteoclasts versus 0.02 or below elsewhere; osteoclast versus next-highest compartment Cohen’s d = 0.36, 95% CI = 0.34–0.38), including above the osteoblast-like tumor and epithelial cells of osteosarcoma (d = 0.51, 95% CI = 0.49–0.52; Figure 4G). The podosome module was higher in tumor, epithelial, and endothelial cells than in osteoclasts, as expected for cytoskeletal machinery shared by invasive cells, and we therefore read it as motility machinery that osteoclasts hold in common with other invasive cells; the transcription-factor module was higher in macrophages. The protease MMP19 did not separate osteoclasts from macrophages (Cohen’s d = 0.03), in line with recent work describing an MMP19- and RANK-positive macrophage rather than an osteoclast-specific MMP19 state (Zhang et al., 2021; Hu et al., 2026a), so we considered MMP19 only a named, non-specific member and reported a specific-core score with such genes removed (STAR methods).

The sulfation module is the most striking part of the program. PAPSS2, EXT1, and FAM20C all act in skeletal matrix biology, spanning sulfation, heparan-sulfate-dependent skeletal signaling and biomineralization (Fan et al., 2018; Hirose et al., 2020; Petersen et al., 2025), and to our knowledge, this combination has not been reported as a shared feature of tumor-associated osteoclasts. Because the atlas is generated from dissociated tumor tissue, we assessed the module against a dedicated adult osteoblast comparator, an independent bone-marrow niche atlas with 2,555 author-annotated osteoblasts from 12 donors (GSE253355; Bandyopadhyay et al., 2024). The complete 74-gene program barely distinguished those osteoblasts from their own mesenchymal precursors (paired donor difference 0.030, 95% CI = 0.012–0.047), so the program as a whole does not describe osteoblast differentiation. Within this reference, SLC16A10, one of the two program genes most strongly correlated with doublet score in the atlas, was essentially undetectable in adult osteoblasts (median detection 0.000, highest 0.013, across 12 donors; per-gene detection fractions in Supplementary Table S7) while reaching 0.24 in mature osteoclasts in the atlas, so an osteoclast–osteoblast multiplet is unlikely to be the source of this signal, and PAPSS2 was likewise detected more often in atlas osteoclasts (Figures 4H–K; Supplementary Figure S5). In intact bone, the association between the module and osteoclast markers strengthens once the osteoblast signature is held constant (Supplementary Figure S8). The module therefore sits on the skeletal–matrix axis that bone-resorbing and bone-forming cells share, and in bone-tumor tissue, it is the mature osteoclast that carries it.

### The program is not an artifact of multinucleated cell doublets or of ambient RNA

Multinucleated osteoclasts form physical multiplets more readily than small myeloid cells, and the within-tumor comparison reduces ambient RNA but not doublets, so we tested the program against doublet load directly. Within osteoclasts, program score and doublet score were essentially uncorrelated (Spearman 0.02); removing the most doublet-prone decile of osteoclasts left the osteoclast-over-macrophage enrichment unchanged (Cohen’s d = 1.08, 95% CI = 1.06–1.10; Figure 4F); and a specific-core score that excluded MMP19 and the biologically implausible ACTN2, a striated-muscle actinin, and COL27A1, a fibrillar collagen, gave essentially the same result (d 1.03, 95% CI 1.01–1.05; Figure 4F). Program-high osteoclasts exhibited only a slightly higher doublet score than the remaining (0.125 versus 0.096). A deliberately conservative filter aimed at osteoclast–osteoblast multiplets, which additionally removed every cell scoring in the top decile of both canonical signatures, retained 83.3% of mature osteoclasts per donor and left the donor-level program score essentially unchanged (−0.025, 95% CI = −0.033 to −0.017; Figure 4L; Supplementary Figure S3).

Doublet filtering removes whole cells; ambient RNA requires a model. We applied the official decontX generative model to each of the 88 mapped sequencing libraries separately, with the published defaults unchanged, and pooled technical libraries into 72 biological donors (STAR methods) (Yang et al., 2020). All 88 donors converged, and after correction, the program remained higher in mature osteoclasts than in macrophages in 58 of 72 donors (donor median 0.137, bootstrap 95% CI = 0.092–0.176; P = 1.4 × 10^−9^); the same pattern was observed for the four-gene sulfation module (median 0.203, 95% CI 0.145–0.302), with UST, EXT1, and RFX8 each meeting the prespecified support rule (Supplementary Figure S13). The ambient contribution estimated by a formal generative model from these libraries therefore does not account for the program.

### The program increases through osteoclast differentiation and tracks mature-osteoclast abundance in intact bone

The atlas analyses are cross-sectional, so we tested the program where osteoclast formation is driven experimentally and sampled over time. An independent bulk RNA-sequencing series of M-CSF- and RANKL-driven differentiation of human monocytes into osteoclasts, eight donors at days 0, 2, 5, and 9, supplied that test on a different platform and in a culture containing neither osteoblasts nor tumor stroma (GSE246769; Hansen et al., 2024). Of the 73 measurable program genes, 52 increased significantly by day 9 (Benjamini–Hochberg FDR <0.05). In donor-blocked models, the program score increased steadily with time (β = 0.163 per day, 95% CI = 0.143–0.184; P = 1.3 × 10^−12^), in step with the canonical maturation score, and the sulfation and mineralization module increased with them (β = 0.143 per day, 95% CI 0.100–0.185); UST, EXT1, PAPSS2, SLC16A10, and the candidate regulator RFX8 were each individually induced (Figures 3I,J; Supplementary Figure S4). Because this culture contains a single differentiating lineage and no bone-forming or tumor cells, the induction of the sulfation enzymes and of RFX8 does not depend on the presence of an osteoblast or tumor source and is most simply interpreted as part of osteoclast formation. On this evidence, the program, along with the matrix-sulfation module within it, behaves as a transcriptional feature that human osteoclasts acquire as they mature.

**FIGURE 5:**
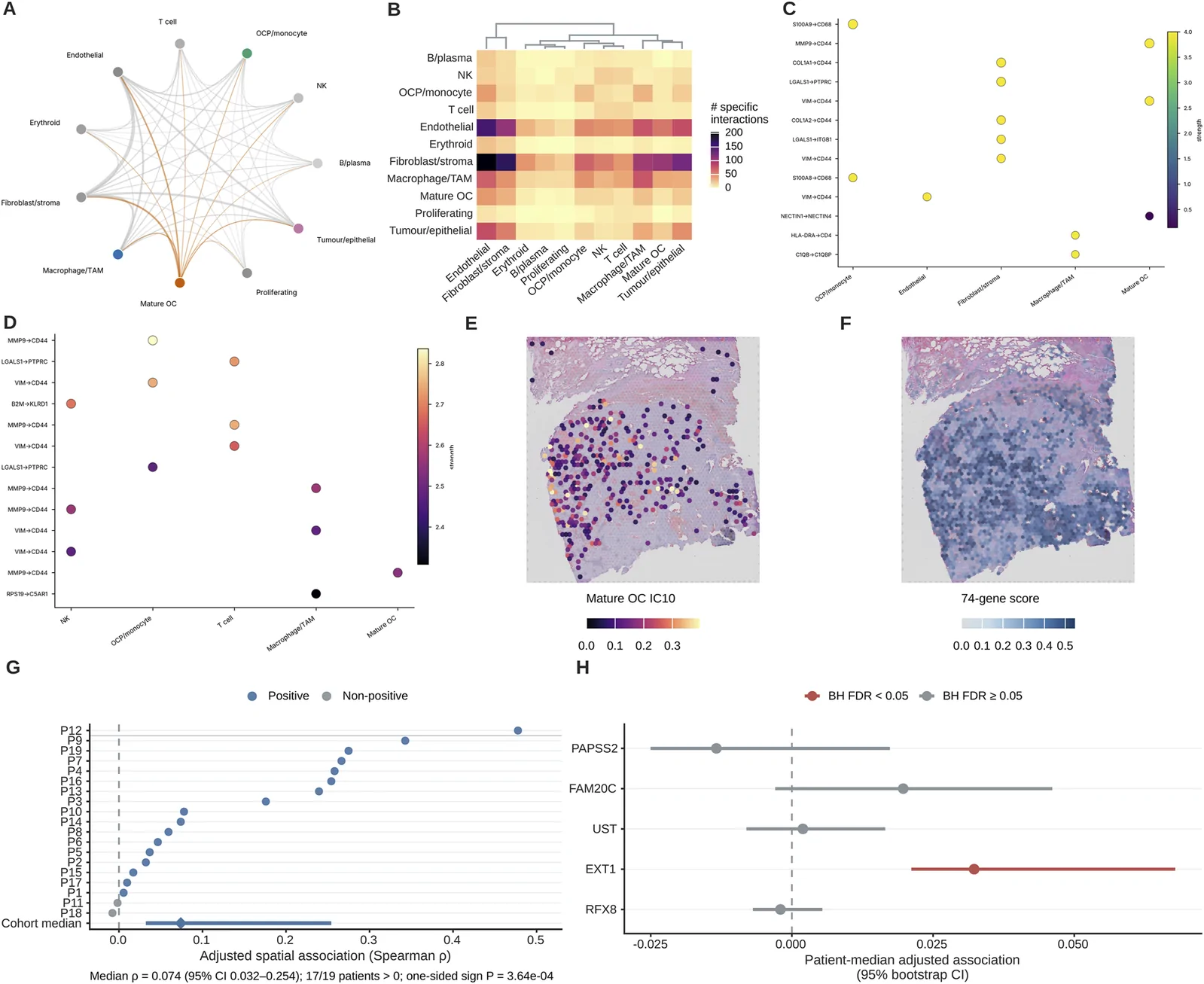
Osteoclasts are associated with a matrix-adhesion signaling neighborhood, and an independent spatial cohort corroborates its association with mature-osteoclast-rich regions in intact tumor tissue. **(A)** Inferred ligand–receptor signaling among compartments (LIANA; chords weighted by interaction strength; osteoclast highlighted). **(B)** Aggregated interaction-strength matrix (clustered; number of specific interactions). **(C)** Top ligand–receptor pairs predicted to signal to osteoclasts, by source compartment (dot color, interaction strength). **(D)** Top pairs predicted to signal from osteoclasts, by target compartment. **(E)** Source-derived mature-osteoclast composition in an illustrative GSE293065 section. **(F)** Locked 74-gene program score in the matched section. **(E, F)** show a representative section (MR4) chosen for display only by a recorded coverage-and-distance rule; all inference is patient-level across all 19 patients. **(G)** Adjusted patient-level program effects for all 19 patients with cohort median, bootstrap CI, and one-sided sign test (17 of 19 positive; median 0.074). **(H)** Patient median effects and Benjamini–Hochberg status for the five prespecified candidate genes (EXT1 FDR = 0.011); robustness analyses in Supplementary Figure S12.

Dissociation also removes cells from their tissue context, so we asked whether the program could be detected where osteoclasts actually reside, using an independent human osteosarcoma spatial transcriptomic cohort comprising 26 Visium sections from 19 patients that contributed to no earlier step (GSE293065; Moquin-Beaudry et al., 2026; STAR methods). Within each section, the program score was related across spots to the mature-osteoclast composition derived by the original authors, adjusted for library size, stromal composition, and a prespecified osteoblastic and chondroblastic tumor composition, and aggregated to one effect per patient. The program tracked mature-osteoclast abundance in 17 of 19 patients (patient median = 0.074, bootstrap 95% CI = 0.032–0.254; one-sided sign P = 3.6 × 10^−4^; Figures 5E–G), and the association held after removing the 11 genes that overlap the authors’ own osteoclast component (16 of 19 patients; Supplementary Figure S12). Among the prespecified candidates, EXT1 showed the individually significant spatial association after correction (FDR = 0.011; Figure 5H). A Visium section of normal human femoral head yielded the same result, and in that tissue, the correlation between the sulfation module and the osteoclast-marker score increased once the osteoblast signature was held constant (Supplementary Figure S8) (Lin et al., 2025). A program defined in dissociated single cells therefore corroborates its association with mature-osteoclast-rich regions in intact human bone, at spot rather than single-cell resolution, measured on a different platform.

### The program reproduces across cancers of origin and in two external cohorts

A shared state should reproduce beyond the tumors that defined it, on axes independent of the selection comparison. Within the bone-metastasis atlas, the osteoclast program score was increased across every cancer of origin tested: breast, colon, lung, and renal cancers (median scores 0.60 to 0.73 against a near-zero background; Figure 4D), so the convergence is not the work of a single primary tumor. We then scored the program, without any tuning, in two cohorts held out of every prior step. In an independent osteosarcoma cohort of 11 tumors (130,657 cells), osteoclasts scored above non-osteoclasts by a small and consistent margin (Cohen’s d = 0.38, 95% CI = 0.36–0.39; 38,361 osteoclasts; Figure 4E), as expected given that the comparator pool itself contained osteoclast progenitors and macrophages. Most directly, a prostate bone-metastasis cohort allowed osteoclasts to be compared head-to-head with tumor-associated macrophages from the same tissue, a contrast that the selection step had never seen, and the separation was large (Cohen’s d 2.74, 95% CI 2.39 to 3.07; 262 osteoclasts against 15,205 macrophages). Viewed by effect size, the modest separation observed with a mixed comparator versus the large separation observed with a pure macrophage comparator is the pattern expected from an osteoclast-enriched program. As expected for a signal confined to a rare cell type, whole-tissue averages showed no difference. Independently, 70%–76% of the program genes re-emerged as osteoclast-enriched in the held-out cohorts (Supplementary Figure S2). These held-out analyses establish reproducibility across datasets and platforms; the differentiation series above and the spatial cohort below carry the evidence into an experimental culture and into intact tissue. The external datasets contributed in distinct roles: GSE152048 as an osteosarcoma replication cohort, GSE266330 as a bone-metastasis ecosystem context, and OEP005136 as a future pan-cancer benchmark (Li et al., 2024; Chu et al., 2025; Wang et al., 2026).

### Osteoclasts are associated with a matrix-adhesion signaling neighborhood

Having localized the program, we asked which signals surround the cells that carry it by inferring ligand–receptor communication among the 11 compartments (Figures 5A–D; STAR methods). Mature osteoclasts resided centrally in the inferred network, not at its edge (Figures 5A,B). The signals they were predicted to receive came from the matrix and adhesion cues sent by the fibroblast and stromal compartment: TIMP1-CD63, collagen-I (COL1A1/COL1A2)-CD44, galectin-1 (LGALS1)-integrin-β1, and vimentin-CD44 (Figure 5C). The signals they were predicted to send were led by the matrix protease MMP9 and by galectin-1 acting on CD44 and PTPRC on lineage and lymphoid cells (Figure 5D). This CD44- and integrin-centered communication is computational inference, consistent with the matrix-remodeling phenotype described by the program, and it places the tumor osteoclast in association with the collagen-rich stroma. Canonical RANKL–RANK signaling remains central to osteoclast biology and complements this picture: the receptor TNFRSF11A/RANK was detected more frequently in mature osteoclasts than in any other compartment, whereas its ligand TNFSF11 is barely captured by droplet chemistry, and the osteoblast-lineage cells that present RANKL are under-represented in dissociated tumor tissue (Supplementary Figure S10); thus, the absence of a RANKL–RANK pair from the inferred network reflects what this assay can detect.

### Canonical regulators and a maturation trajectory place the program at the osteoclast terminus

**FIGURE 6:**
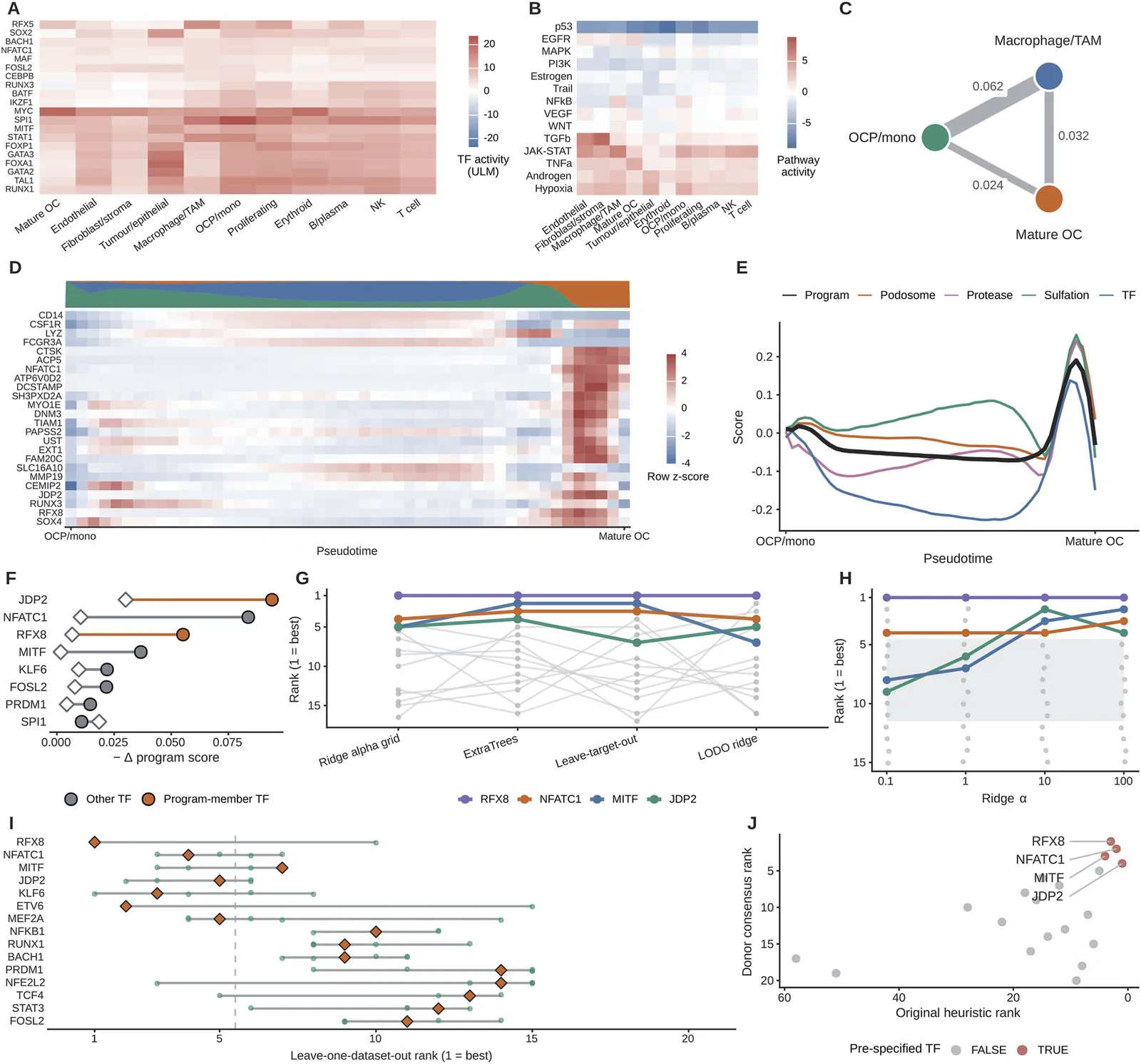
Canonical regulators, pathway activity, and a maturation trajectory place the program at the osteoclast terminus. **(A)** Transcription-factor activity per compartment (DoRothEA ULM; clustered, RdBu_r). **(B)** Pathway activity per compartment (PROGENy MLM; clustered). **(C)** PAGA lineage abstraction (node, compartment; edge width, connectivity). **(D)** Pseudotime-ordered gene-expression heatmap (row z-score; top bar, compartment composition along pseudotime); osteoclast markers and program genes increase toward the mature-osteoclast end. **(E)** Program and module scores along pseudotime (line, mean; band, s.e.m.). **(F)** In silico knockout (lollipop; filled, -Δ program for the real network; open diamond, factor-specific shuffled null; program-member factors flagged). **(G)** Multi-method consensus rank trajectories for all candidate factors. **(H)** Ridge-penalty rank paths across α = 0.1, 1, 10, and 100. **(I)** Leave-one-dataset-out ranks for each factor. **(J)** Original heuristic rank against the donor-level consensus rank; sensitivity details in Supplementary Figure S7.

We next set the program in its regulatory and developmental context. Transcription-factor activity inferred per compartment recovered the canonical osteoclast regulators PU.1/SPI1, MITF, NFATc1, and FOSL2 among the active factors of the lineage (Figure 6A), confirming osteoclast identity through a route independent of the program score. Pathway-activity inference placed TNF-α and hypoxia signaling highest in mature osteoclasts (Figure 6B), in keeping with RANKL and TNF-superfamily input and the hypoxic microenvironment of active resorption. Abstracting the lineage recovered the expected osteoclastogenic topology, with an osteoclast-progenitor and monocyte compartment connected to both a macrophage fate and the mature osteoclast (Figure 6C); ordering cells along a diffusion pseudotime from the progenitor root showed the canonical maturation markers CTSK, ACP5, and ATP6V0D2 increasing toward the mature end, with the program genes following them (Figures 6D,E), recapitulating the differentiation series observed directly over time (Figures 3I,J). The program is therefore a terminal feature of osteoclast differentiation, not a property of a side population.

Within this regulatory map, we asked which factors are predicted to regulate the program by linking candidate transcription factors to it and simulating *in silico* knockouts, each benchmarked against its own degree-preserving shuffled-network null (Figure 6F; STAR methods; Equations 5 and 6). The clearest case is NFATc1, the master regulator of osteoclastogenesis and not itself a program gene: removing it collapsed the program (Δ −0.084, 8.0-fold over its own null), showing that the analysis recovers known biology (Kawaida et al., 2003). Two of the top-ranked factors, JDP2 (Δ −0.094, 3.1-fold) and RFX8 (Δ −0.055, 8.2-fold; highest program correlation, r = 0.69), are themselves program genes, so each was re-scored with itself excluded from its own outcome. Across four ridge penalties, a second model class, 2,000 permutations per factor, and seven leave-one-dataset-out repetitions, RFX8 ranked the first on every consensus, followed by NFATc1, MITF, and JDP2 (Figures 6G–J; Supplementary Figure S7). RFX8 has no prior reported role in osteoclasts or bone and is induced as human monocytes differentiate into osteoclasts, which makes it the most novel candidate the program nominates; these analyses are observational and generate hypotheses for experimental testing, with the most tractable nodes they identify being the sulfation enzymes PAPSS2, FAM20C, and UST.

### The program is elevated in tumor osteoclasts against a matched adult control

To weigh the program against normal adult bone rather than the developmental reference used for discovery, we compared tumor osteoclasts with non-tumor osteoclasts from control bone in the bone-metastasis cohort on the same platform (STAR methods). The program remained increased in tumor osteoclasts in every group, including the held-out osteosarcoma (AUROC 0.83 to 0.91; Supplementary Figures S2B,D), and held at the sample level, with 96% of 53 tumor samples exceeding the control (Supplementary Figure S2E). With the donor as the unit, the difference remained large (Hedges’ g = 1.434, 95% CI = 1.155–1.883; 27 tumor and 3 adult-control donors; Figure 3F), and the same framework showed the program moving with osteoclast maturation, recapitulating the differentiation series measured directly (Figures 3H–J). With the donor-level maturation score held constant, the residual tumor coefficient was small (0.045, 95% CI = −0.058 to 0.149; n = 30 donors), and the state is therefore described throughout as a maturation-associated osteoclast program reproducibly enriched in bone-tumor tissue. Functional enrichment of the 74 genes (Supplementary Figure S2A) was led by estrogen- and androgen-response and hypoxia programs, implicating hormone- and oxygen-sensing axes with established roles in bone remodeling, consistent with a state-level rather than single-marker signature.

As an expected-direction control, osteoclasts from denosumab-treated giant-cell tumors exhibited a lower program score and were depleted from the lineage (osteoclast fraction 0.338 down to 0.130 across two treated and six untreated samples), providing the program face validity against anti-RANKL therapy in the direction that osteoclast biology predicts (Supplementary Figure S1). The program is thus best described as a tumor-associated, maturation-linked osteoclast state, and a more deeply sampled adult reference is the natural way to sharpen that boundary further.

## Discussion

Across three classes of human bone tumor, tumor osteoclasts share a definable, reproducible transcriptional program. It is not explained by contamination or by myeloid identity: it survives formal ambient-RNA modeling, conservative multiplet filtering, and comparison with an adult osteoblast reference, it reproduces in tumors and cohorts it had never seen, it increases through human osteoclast differentiation, and it tracks mature-osteoclast abundance in intact bone, at spot rather than single-cell resolution. Its osteoclast-enriched core is a matrix-sulfation and mineralization module. The work also offers a methodological lesson. In tumor tissue, a tumor-versus-normal comparison is uninterpretable without a within-tissue control, and the same naive analysis that produced a 1,703-gene contamination signature here is the default across much of the single-cell literature.

Recent work already supports osteoclast involvement in GCTB, osteosarcoma, and bone metastasis; what has been missing is a mature-osteoclast state that is conserved across tumor types and survives testing against macrophage, ambient RNA, doublet, and maturation confounders (Zhang N. et al., 2024; Liu J. et al., 2025; Chandrasekaran et al., 2026; Kottmann et al., 2026; Zehenter et al., 2026a).

The program combines established osteoclast biology with new ground. The canonical anchors NFATc1, CTSK, ACP5, and ATP6V0D2, the podosome and actin-motility machinery, hypoxia signaling, and broad extracellular-matrix remodeling all behave here as mature-osteoclast biology should, and recovering them is evidence that the analysis captured the intended cell. What this study adds is the cross-disease convergence of one bounded, osteoclast-high, and myeloid-low 74-gene set and, within its narrower osteoclast-enriched core, two elements for which we found no precedent in osteoclast biology: the glycosaminoglycan-sulfation and mineralization module built from PAPSS2, UST, EXT1, and FAM20C, and the nomination of RFX8. The remaining modules are shared with other cell types, and the much-discussed protease MMP19 does not separate osteoclasts from macrophages, in line with reports that the MMP19-positive bone-metastasis cell is a macrophage (Zhang W. et al., 2024; Zhu et al., 2025); the same context-dependence applies to SPP1, CSF1R, and FAM20C (Zuo et al., 2021b; Su et al., 2025; Hu et al., 2026a; Liang et al., 2026; Zehenter et al., 2026b), which is why the sulfation module was tested outside the atlas. Working the other way, sulfate-biology genes and heparan-sulfate interactions with cathepsin K offer a plausible matrix-remodeling mechanism for this effect (Patel et al., 2018; Zhu et al., 2022; Lee et al., 2025). We therefore consider the sulfation module the sharpest hypothesis from this work, that tumor-associated mature osteoclasts upregulate matrix-sulfation machinery as part of their resorptive program.

Among the gene-level candidates, RFX8 is the most novel. RFX-family studies define its regulatory context, and pan-cancer work implicates RFX8 in leukemia, but we found no prior evidence tying it to osteoclast differentiation, GCTB, osteosarcoma, bone metastasis, or denosumab response (Sugiaman-Trapman et al., 2018; Cui et al., 2024). Here, it is predicted to regulate the program across every model class, penalty, permutation, and dataset omission tested, and it is induced as human monocytes differentiate into osteoclasts. RFX8 is thus a nominated candidate regulator of the osteoclast program, supported by observational and *in silico* analyses rather than demonstrated as a driver, and awaiting the perturbation experiments that would establish its role.

Three independent analyses support this picture without leaning on the program score: inferred communication places the osteoclast in a CD44- and integrin-centered matrix-adhesion neighborhood fed by collagen-rich stroma, transcription-factor activity recovers PU.1, MITF, and NFATc1 as active in these cells, and a diffusion-pseudotime trajectory places the program at the terminus of osteoclastogenesis. Here, we set out more fully what we have measured and what we put forward as hypotheses. The program itself, its enrichment over the myeloid cells of the same donor, its persistence after ambient-RNA modeling and multiplet filtering, its position relative to an adult osteoblast reference, its increase through human osteoclast differentiation, and its association with mature-osteoclast abundance in intact bone are all measurements. The ligand–receptor interactions around the osteoclast, along with the nomination of RFX8 and the sulfation enzymes as regulators and effectors of the state, are inferences drawn from those measurements, and we consider them hypotheses for experiment to test.

The clinical reach of the program is best pursued as testable directions. Whether it tracks osteolytic activity is measurable against serum TRACP5b and radiographic cortical destruction in the same patients, and the denosumab comparison points that way since anti-RANKL treatment lowered both osteoclast abundance and program score. Whether it carries information about response to denosumab, about prognosis, or about the separation of aggressive from indolent disease are questions for pre-treatment, outcome-annotated cohorts linked to recurrence, survival, Campanacci grade, and MSTS score and are posed here as hypotheses. Whether any program gene is druggable begins with the enzymatic members of the sulfation module, UST, PAPSS2, EXT1, and FAM20C, protein classes that are, in principle, addressable by small molecules; a tractability audit of the program genes is provided in Supplementary Table S11. The literature supplies the setting for each test. In GCTB, denosumab exposure and withdrawal are linked to osteoclast-like giant-cell depletion and rebound, neoplastic-cell state shifts, Treg and TREM2-positive macrophage remodeling, and CSF1R-sensitive recurrence, while serum TRACP5b, Campanacci grade, cortical destruction, MSTS score, and pulmonary-metastasis surveillance supply measurable endpoints (Ahmed Kamel et al., 2026; Hu et al., 2026b; Toda et al., 2026; Uzun et al., 2026; Yao et al., 2026). In osteosarcoma, the osteoclast axis connects to ITGA2, PPARG, and cisplatin-resistant osteoclast-precursor maturation (Geng et al., 2024; Jiang et al., 2024; Wei et al., 2025). In bone metastasis, denosumab resistance and osteopontin-producing osteoclasts further mark osteoclast-state biology as clinically relevant, although outcome prediction will need testing in annotated cohorts (Cheng et al., 2025; Lin et al., 2026).

### Limitations of the study

Several boundaries define the next experiments. Everything reported here comes from human data that already exist, so prospective validation in newly collected, outcome-annotated tissue is the step that would carry the program from a reproducible signature toward a clinical tool. The adult osteoclast control on the same platform is small (134 osteoclasts from three donors), and a deeply sampled adult and *in vitro* resource would sharpen the developmental comparison; the differentiation series analyzed here is a first instalment of it. Tissue dissociation is selective against large multinucleated cells and can generate heterotypic multiplets, which is why the program was tested against conservative multiplet filtering, formal ambient-RNA modeling, and two spatial platforms that leave the tissue intact. These platforms measure many cells per spot, so cell-resolved co-localization with CTSK and ACP5 by immunohistochemistry or RNA *in situ* hybridization is the assay that would place the program inside individual osteoclasts. The atlas is cross-sectional, so its ordering along maturation is an association; the differentiation series supplies the temporal axis directly, and longer time courses with perturbation would resolve the order in which the modules switch on. The external osteoclast definitions are marker-based, the direct prostate comparison rests on 262 cells, the RFX8 nomination is observational, and the denosumab read-out is specific to GCTB.

The most direct next steps are spatial and functional rather than another single-cell reanalysis. Pathology in GCTB and osteosarcoma already uses FOS, JDP2, and NFATc1 immunohistochemistry, multiplex imaging, and ECM-aware analyses to distinguish osteoclast maturation from histiocyte and TAM patterns (Agawa et al., 2024; Gomez-Mascard et al., 2024; Yang et al., 2024). A pathology-compatible assay should co-localize RFX8 or sulfation-module genes with CTSK and ACP5 and assess their exclusion from CD68-, CD163-, and TREM2-rich macrophages, in sections linked to recurrence, denosumab exposure, cortical destruction, MSTS score or TRACP5b, and functional testing of RFX8 and the sulfation enzymes in human osteoclast cultures. The defining resource for this program is deeper osteoclast and osteoblast sampling together with those experiments.

## STAR methods

### Key resources table

**Table:** 

Reagent or resource	Source	Identifier
Bone metastasis scRNA-seq (+ adult control bone)	NCBI GEO	GSE266330
Giant-cell tumor of bone scRNA-seq	NCBI GEO	GSE254672
Giant-cell tumor of bone scRNA-seq	NCBI GEO	GSE168664
Osteosarcoma scRNA-seq	NCBI GEO	GSE162454
GCTB ± denosumab scRNA-seq	NCBI GEO	GSE268835; GSE212341
Osteosarcoma scRNA-seq (external)	NCBI GEO	GSE152048
Prostate bone-metastasis scRNA-seq (external)	NCBI GEO	GSE143791
Osteoclast reference (fetal)	CELLxGENE census	census
scanpy	(Wolf et al., 2018)	RRID:SCR_018139
scvi-tools	(Lopez et al., 2018)	RRID:SCR_022698
LIANA+ (cell-cell communication)	(Dimitrov et al., 2024)	RRID:SCR_026620
decoupler (TF/pathway activity)	Badia-I-Mompel et al. (2022)	RRID:SCR_022687
Enrichr/GSEApy	(Kuleshov et al., 2016)	RRID:SCR_001575
Custom analysis code	This paper	https://github.com/tars9002-create/Osteoclast; commit 88005b4
Human osteoclast differentiation bulk RNA-seq	NCBI GEO	GSE246769
Adult bone-marrow niche scRNA-seq (osteoblast reference)	NCBI GEO	GSE253355
Osteosarcoma spatial transcriptomics (Visium)	NCBI GEO/Moquin-Beaudry et al., 2026	GSE293065
Normal femoral-head spatial transcriptomics (Visium)	NCBI GEO	GSE284089
decontX (ambient-RNA modelling)	(Yang et al., 2020)	RRID:SCR_024462
edgeR (bulk differential expression)	Bioconductor	RRID:SCR_012802
Seurat (external reference handling)	Bioconductor/CRAN	RRID:SCR_016341

## Method details

### Cohorts and integration

We analyzed open human droplet single-cell RNA-seq from the NCBI GEO and the CELLxGENE census (Table 1). Where a repository did not state the exact chemistry, we describe the platform as droplet rather than infer it. Raw count matrices were size-checked against repository manifests, hashed (sha256), confirmed to contain integer counts, and loaded with scanpy (Wolf et al., 2018). Discovery and replication cohorts were assigned with zero sample overlap, and the replication and external cohorts (GSE152048, GSE143791) were evaluated once. Dataset roles were pre-specified: discovery and held-out-disease cohorts for program derivation and replication; GSE152048 as an osteosarcoma external replication cohort; GSE143791 as a prostate bone-metastasis osteoclast-versus-TAM comparison; GSE266330 as a bone-metastasis ecosystem reference; GSE246769 as an independent human osteoclast-differentiation series; GSE253355 as an adult osteoblast-lineage reference; GSE293065 as an independent human osteosarcoma spatial cohort; GSE284089 as a normal-bone spatial reference; and OEP005136/HRA016527 as high-priority validation resources for pan-cancer bone metastasis and for denosumab-treated or recurrent GCTB (Hu et al., 2026b; Wang et al., 2026). Cells were filtered (≥200 genes, ≥500 counts, <25% mitochondrial reads), and doublets were scored with scrublet. Counts were harmonized to Ensembl identifiers (29,337 shared genes), integrated with scVI (n_latent = 30) (Lopez et al., 2018), clustered with Leiden (resolution 2.0), and annotated to 11 compartments by canonical markers, with a guard that called a cluster mature osteoclast only when its osteoclast signature exceeded both stromal and osteoblast signatures. The osteoblast signature used as that guard was assembled from the canonical markers of human osteoblast differentiation used in bone single-cell studies: the master transcription factors RUNX2 and SP7, the matrix and mineralization markers ALPL, BGLAP, and IBSP, the osteoid collagens COL1A1, COL1A2, and COL3A1, and the osteoblast-associated markers SPP1 and SATB2. It was scored as a ten-gene panel since several of these transcripts, SPP1 in particular, are also expressed in tumor and myeloid contexts, and it was applied conservatively as a guard against misannotation. The complete compartment key, with positive-marker panels, guards, cell counts and display colors, is provided in Supplementary Table S1. In external cohorts, osteoclasts were defined by CTSK and ACP5 co-expression and tumor-associated macrophages by a CD68/C1QA/CD163/LYZ score.

### Ambient model and the interaction filter

We model the observed log-normalized expression $x_{cg}$ of gene g in cell c , in compartment $k\left(c\right)$ within tumor $t\left(c\right)$ , as a compartment-intrinsic mean plus a tumor-wide ambient term shared by co-dissociated compartments and noise:

$$x_{cg}=\mu_{k\left(c\right)\;g}+a_{t\left(c\right)\;g}+\varepsilon_{cg}.$$

A naive tumor-versus-reference osteoclast contrast then confounds the biological difference with the difference in ambient load:

$$\Delta_{g}^{\text{naive}}=\left(\mu_{\text{OC},g}^{\text{tum}}-\mu_{\text{OC},g}^{\text{ref}}\right)+\left(a_{g}^{\text{tum}}-a_{g}^{\text{ref}}\right),$$

whereas an osteoclast-versus-myeloid contrast *within the same tumor* shares $a_{t\;g}$ and so suppresses it. A gene g entered the program if, in every discovery tumor t , it was upregulated in tumor osteoclasts versus reference osteoclasts and enriched in osteoclasts versus that tumor’s myeloid cells:

$$P=\underset{t}{⋂}\left\{\;g:\Delta_{t,g}^{\text{tum}‐\text{ref}}>\tau_{1}\wedge \left(\mu_{\text{OC},t,g}-\mu_{\text{mye},t,g}\right)>\tau_{2}\;\right\},$$

with $\tau_{1}=0.25$ , $\tau_{2}=0.5$ on $\log_{2}$ fold change (Wilcoxon and Benjamini–Hochberg FDR <0.05 ), after removal of foreign-lineage and technical genes (a fixed exclusion list of erythroid, platelet, immunoglobulin, mitochondrial, ribosomal, globin, heat-shock, immediate-early, and cell-cycle genes; the complete symbol-level list of the 100 foreign-lineage and 307 technical genes present in the 29,337-gene universe, each with the rule that excluded it, is provided in Supplementary Table S3). The within-tumor osteoclast-versus-myeloid criterion substantially reduces the shared ambient component, and the residual contribution is estimated separately with a formal generative model below, so the genes that satisfy both criteria are osteoclast-enriched.

### Program score and specificity

For a gene set P , the per-cell program score is the scanpy score_genes statistic:

$$s_{c}=\frac{1}{\left|P\right|}\sum_{g\in P}x_{cg}-\frac{1}{\left|R\right|}\sum_{g\in R}x_{cg},$$

where R is an expression-matched control set (ctrl_size 50, 25 bins, seed 0). Compartment and group specificity were quantified by Cohen’s d between osteoclasts and each comparison compartment k :

$$d_{k}=\frac{{\bar{s}}_{\text{OC}}-{\bar{s}}_{k}}{s_{\text{pooled}}},$$

with 1,000-sample bootstrap 95% confidence intervals reported for every d . Module scores apply Equation 4 to each module’s genes; a “specific-core” score excludes the non-specific genes MMP19, ACTN2, and COL27A1. Read-depth confounding was controlled by residualizing $s_{c}$ on $\log_{10}$ total counts, maturation confounding by residualizing on a canonical maturation score, and doublet confounding by per-gene regression on doublet score and by re-deriving effects after removing the top doublet-score decile of osteoclasts. Reproduction was assessed using AUROC against adult and fetal references and by the fraction of program genes re-recovered in held-out cohorts.

### In silico knockout

Candidate transcription-factor regulators were linked to program genes by ridge regression of the program-gene matrix S on transcription-factor expression X across osteoclast-lineage cells, $\hat{W}=\arg\min_{W}∥S-XW{∥}_{F}^{2}+\lambda ∥W{∥}_{F}^{2}$ . Knockouts were simulated by setting a factor to zero and propagating the change through $\hat{W}$ for three steps, reimplementing the CellOracle propagation without building on its architecture (Kamimoto et al., 2023); the effect was reported as a raw change and a factor-specific null-calibrated score:

$$\Delta s=\bar{s\left({\hat{X}}^{\text{KO}}\right)}-\bar{s\left(X\right)},\;z=\frac{\Delta s-\mu_{\text{null}}}{\sigma_{\text{null}}},$$

with $\mu_{\text{null}},\sigma_{\text{null}}$ estimated per factor over degree-preserving shuffled networks. Knockout effects for factors that are themselves program genes are partly self-referential.

### Assumptions, parameter robustness, and comparison with established methods

The regulatory analysis is observational and rests on three assumptions, which we state explicitly. First, co-variation between a transcription factor and the program genes across cells is taken to indicate a regulatory relationship, although co-variation alone carries no direction. Second, the ridge coefficients estimated on the observed gene universe are taken to approximate the consequence of removing a factor, although both the gene universe and the regularization strength influence the ranking. Third, transcript abundance is taken to approximate regulator activity, although it does not measure protein abundance, nuclear localization, or promoter occupancy. Because a factor that is itself a program gene contributes to its own outcome, every added estimate excluded each factor from the outcome against which it was scored and from the predictor set for its own target. Robustness to parameter selection was assessed over ridge penalties α = 0.1, 1, 10, and 100; robustness to model class by refitting with a 500-tree ExtraTrees ensemble per target; statistical calibration by 2,000 degree-preserving permutations per factor; and robustness to dataset composition by seven leave-one-dataset-out repetitions. RFX8 ranked first on the consensus of all of these analyses and first again under the ExtraTrees ensemble; across the 52 donors, its expression tracked the mean expression of the program genes with RFX8 itself removed from that mean (Spearman r = 0.723; 2,000-permutation P = 5.0 × 10^−4^). The propagation step follows the logic introduced by CellOracle, in which a fitted linear model is used to propagate a simulated perturbation through a gene network (Kamimoto et al., 2023); it differs from a CellOracle run in that the network here is inferred from expression alone, whereas CellOracle builds a motif-informed, state-specific base network from chromatin accessibility and couples it to an explicit state-transition model. Because no matched scATAC-seq is available for these cohorts, we treated the multi-model, permutation-calibrated, and leave-one-dataset-out consensus described above as the robustness evidence for the ranking. Simulating the deletion of a transcription factor in observational data cannot reproduce the compensation, feedback, and chromatin remodeling that follow a real perturbation, so these analyses rank candidates for experimental testing.

### Cell–cell communication, regulatory activity, trajectory, and enrichment

Ligand–receptor communication among compartments was inferred with LIANA’s rank-aggregate consensus on a compartment-balanced subsample, reporting magnitude and specificity ranks and an aggregated count of specific interactions per compartment pair (Dimitrov et al., 2024). Transcription-factor activity (DoRothEA, confidence A–C) and pathway activity (PROGENy) were estimated per cell using decoupler’s univariate and multivariate linear models, respectively, and averaged by compartment (Badia-I-Mompel et al., 2022). Lineage structure was abstracted with PAGA on the scVI neighbor graph; a diffusion pseudotime was computed from an osteoclast-progenitor root, and program and marker genes were averaged in 50 equal-cell pseudotime bins for the ordered heatmap (row z-score) and the trend curves. Gene-set enrichment of the 74 genes used Enrichr against GO Biological Process, Reactome, and MSigDB Hallmark (Kuleshov et al., 2016). All procedures used fixed seeds.

### Donor hierarchy and donor-level analysis

Library, sample, and derived-donor identifiers were retained separately, and libraries sharing a donor prefix and differing only by a technical suffix were collapsed before any donor-level test. For each donor and compartment, cell-level scores were averaged after requiring a minimum of 20 cells. The primary lineage contrast was the paired within-donor mature-osteoclast minus myeloid mean, evaluated using a two-sided paired Wilcoxon test and 10,000 donor-level bootstrap resamples. The leave-one-dataset-out analysis repeated that contrast seven times, omitting one accession each time; mean paired differences ranged from 0.199 to 0.229, and every donor was positive in every run.

### External human osteoclast differentiation

Processed GSE246769 bulk RNA-seq comprised eight donors at days 0, 2, 5, and 9 (29 libraries) during M-CSF- and RANKL-driven differentiation of human monocytes into osteoclasts. Seventy-three of the 74 program genes were present after filtering. Scores were means of within-gene standardized log expression. Donor-blocked linear models included time and donor fixed effects, and nested-model F tests compared the time model with the donor-blocked null, donor entering as a fixed block, the specification appropriate to eight donors scored on gene-wise standardized values. Gene-level day-9-versus-day-0 contrasts used edgeR with TMM normalization and Benjamini–Hochberg correction; UST (log2 fold change 2.81, FDR 2.9 × 10^−5^), EXT1 (2.05, FDR 2.7 × 10^−4^), PAPSS2 (1.00, FDR 0.020), and RFX8 (1.83, FDR 8.4 × 10^−4^) were among the genes induced, and the complete gene-level table is provided in Supplementary Table S6.

### External adult osteoblast-lineage reference

The official GSE253355 MSC-subset Seurat object was analyzed without reannotation. Published level-2 labels defined 2,555 osteoblasts and their Osteo-MSC comparators among 19,257 cells from 12 donors. Program and sulfation scores were summarized by donor and annotation and compared as paired donor differences. Candidate-gene detection fractions were compared descriptively between the external osteoblasts and atlas mature osteoclasts, with PAPSS2, SLC16A10, and ST3GAL6 detected in a larger fraction of atlas mature osteoclasts than of adult osteoblasts (median detection 0.32, 0.24, and 0.49 against 0.14, 0.00, and 0.08); absolute normalized values were not compared across platforms.

### Spatial transcriptomics

The GSE293065 human osteosarcoma Visium cohort was analyzed from the deposited count matrices, spot coordinates, and the authors’ own annotation of cellular composition. Barcodes were matched exactly, without fuzzy matching, retaining 53,083 of 54,588 spots across 26 sections from 19 patients; 71 of the 74 program genes were present in every section. Mature-osteoclast abundance was taken as the source-derived IC10 composition, with IC_10, IC_38, IC_68, and IC_143 as the prespecified sensitivity set; the cohort, donor, and library map are provided in Supplementary Table S2, and the per-analysis result tables presented in Supplementary Tables S5, S7-10, 12. Within each section, the association between program score and osteoclast composition was adjusted for log1p total UMI, total stromal composition, and a prespecified osteoblastic and chondroblastic tumor composition; section estimates were aggregated to one effect per patient, and patient effects were summarized by median with 10,000 patient bootstrap intervals and a one-sided sign test. A prespecified sensitivity analysis removed the 11 program genes that overlap the source osteoclast contributive lists, retaining 63 genes (median 0.051, 95% CI 0.017–0.124; 16 of 19 patients). PAPSS2, FAM20C, UST, EXT1, and RFX8 formed one prespecified Benjamini–Hochberg family. Support was prespecified as full when the patient median exceeded zero with at least 75% of patients positive. The sections displayed in Figures 5E,F are illustrative examples chosen by a recorded display rule and carry no inferential weight; random procedures used seed 20260722. The GSE284089 normal femoral-head section was analyzed over 4,992 in-tissue spots, giving for the sulfation module a Spearman correlation with the osteoclast-marker score of 0.174 (5,000-permutation P = 2.0 × 10^−4^), a residual correlation after adjustment for log library size and osteoblast score of 0.331, and gene-level comparison of osteoclast-high spots. Visium spots contain multiple cells, so both spatial analyses relate composition to expression at spot resolution.

### Formal ambient-RNA modeling

We reproduced the official decontX example before applying decontX 1.4.1 to each of the 88 mapped filtered-count libraries separately (Yang et al., 2020). The pre-existing broad compartment annotation was supplied as z, with maxIter = 500, delta = c (10,10), estimateDelta = TRUE, convergence = 0.001, iterLogLik = 10, varGenes = 5,000, dbscanEps = 1, and seed = 12,345. Scores were the mean log1p counts per 10,000 current library counts, calculated before and after correction. Technical libraries were pooled by cell-count weighting within 72 biological donors, and the prespecified primary contrast was mature osteoclast minus macrophage, with osteoclast progenitor as a sensitivity comparator. Median effects received 10,000 donor bootstrap intervals, along with two-sided paired Wilcoxon and sign tests. Support was prespecified as full when the corrected median exceeded zero with at least 75% of donors positive; 97% of the donors positive before correction remained positive after it. PAPSS2, FAM20C, UST, EXT1, and RFX8 formed one prespecified Benjamini–Hochberg family. No library, gene, parameter, or threshold was changed after results were inspected.

### Doublet, threshold, and module-stability sensitivity

Scrublet was run separately within each library on the locked osteoclast-lineage object. The continuous doublet score was used throughout, so that the filter acts on the full ranking of every cell. A conservative retained-cell set removed the top library-specific doublet-score decile, together with cells lying simultaneously in the top decile of the canonical osteoclast and osteoblast scores; donor-level mature-osteoclast scores before and after filtering and the retained fraction were reported. The locked 74 genes were then evaluated across 30 combinations of stricter osteoclast (0.5–1.5) and tumor (0.25–1.5) log-fold-change thresholds, reporting retained counts without re-optimization. Donor-level gene means were clustered with average linkage on 1 − Spearman correlation, and 500 donor bootstrap resamples were used to estimate within-cluster co-assignment stability (median 0.97 and 0.78 for the two multi-gene clusters, which hold 72 of the 74 genes); the four functional labels used in the figures are curated annotations and are reported alongside these data-driven stabilities.

Integration audit. For a computationally tractable audit, up to 2,500 cells per accession were sampled from the locked latent representation (17,500 cells in total). Thirty-nearest-neighbor local batch entropy and subtype purity were calculated, together with accession and subtype silhouette coefficients, following published atlas-integration benchmarks (Luecken et al., 2022): the mean local subtype purity was 0.916, subtype silhouette was 0.114, accession silhouette was 0.006, and mean local batch entropy was 0.350.

## Quantification and statistical analysis

The biological donor is the independent unit for the primary score analyses reported here, and single cells are treated as repeated, correlated observations within donors. Technical libraries were collapsed to donors before any donor-level test; within-donor mature-osteoclast and myeloid means were compared with a two-sided paired Wilcoxon test, with confidence intervals from 10,000 donor-level bootstrap resamples. Tumor-versus-adult-control comparisons used collapsed donor summaries with Hedges’ g and donor bootstrap intervals; GSE246769 used donor-blocked fixed-effect models with nested-model F tests; GSE253355 used paired donor differences; and the spatial cohort used the patient as the unit of analysis, aggregating the sections of a patient before inference. Cell-level statistics are reported as descriptive summaries of the underlying cell distributions.

Differential expression used the two-sided Wilcoxon rank-sum test with Benjamini–Hochberg correction. Group contrasts used Cohen’s d with 1,000-sample bootstrap 95% confidence intervals; those cell-level intervals are narrow because they summarize large numbers of correlated cells within donors, and they are reported as descriptive summaries of the underlying cell distribution, while the donor-, patient-, and population-level claims rest on the donor-level statistics (Squair et al., 2021; Zimmerman et al., 2021); AUROCs are reported with bootstrap confidence intervals and compared with DeLong’s test where compared inferentially. The denosumab contrast used a two-sided sample-level Mann–Whitney test, which at *n* = 2 versus 6 cannot reach significance and is reported as a face-validity check; per-cell tests are pseudoreplicated and are not used. Gene symbols follow current HGNC nomenclature and are set in italic type, protein names are set in roman type with the capitalization conventional for each factor (the gene NFATC1 and the protein NFATc1, for example), and pathway and gene-set names follow their source database. All random procedures used a fixed seed (0), except where a different seed is stated for an analysis added during revision. Analyses used scanpy 1.11 and scvi-tools 1.3 on PyTorch 2.12, together with R 4.4.2, decontX 1.4.1, edgeR, and Seurat for the analyses added during revision. Large language models assisted manuscript drafting and code editing only and did not generate, alter, or interpret data; every value added in this revision was verified against the machine-generated result files, in line with journal policy.

## Data Availability

All single-cell datasets analyzed in this study are publicly available under the accessions listed in the key resources table and Table 1. Derived program signatures, the per-gene evidence and doublet-loading matrices, effect-size tables with confidence intervals, and per-sample values are provided as supplemental tables. Custom analysis code for data discovery, atlas construction, convergent-program discovery, validation, in-silico perturbation, downstream analysis, and figure generation is available at https://github.com/tars9002-create/Osteoclast-revision. The repository contains the analysis scripts, dependency specifications, and reproducibility notes. Raw public datasets, large intermediate single-cell objects, local cluster outputs, manuscript documents, and generated figure files are not tracked in Git and should be regenerated from the public data using the documented workflow.

## References

[B1] Agawa R. Kato I. Kawabata Y. Takeyama M. Fujii S. (2024). Histological and immunohistochemical analyses of osteoclast maturation in giant cell tumor of bone. Pathol. Res. Pract. 254, 155128. 10.1016/j.prp.2024.155128 38244437

[B2] Ahmed Kamel S. Evans S. Etaiwi M. Davies M. Gavvala S. N. Botchu R. (2026). Role of chest imaging in GCTB staging and surveillance. Clin. Imaging 134, 110813. 10.1016/j.clinimag.2026.110813 42019324

[B3] Badia-I-Mompel P. Vélez Santiago J. Braunger J. Geiss C. Dimitrov D. Müller-Dott S. (2022). decoupleR: ensemble of computational methods to infer biological activities from omics data. Bioinform Adv. 2, vbac016. 10.1093/bioadv/vbac016 PMC9710656 36699385

[B4] Bandyopadhyay S. Duffy M. P. Ahn K. J. Sussman J. H. Pang M. Smith D. (2024). Mapping the cellular biogeography of human bone marrow niches using single-cell transcriptomics and proteomic imaging. Cell 187, 3120–3140.e29. 10.1016/j.cell.2024.04.013 PMC11162340 38714197

[B5] Chandrasekaran K. Hu S. Farstad-O’Halloran K. Iyer K. S. Jiang H. Pavlos N. (2026). Nutrients and metabolites as signalling molecules in osteoclasts. Curr. Osteoporos. Rep. 24, 6. 10.1007/s11914-026-00955-4 PMC12886248 41661386

[B6] Cheng J.-N. Jin Z. Su C. Jiang T. Zheng X. Guo J. (2025). Bone metastases diminish extraosseous response to checkpoint blockade immunotherapy through osteopontin-producing osteoclasts. Cancer Cell 43, 1093–1107.e9. 10.1016/j.ccell.2025.03.036 40280123

[B7] Chu J. Qin R. Wang S.-J. Wang Q. Wu Q. (2025). Integrated single-cell and transcriptomic analysis of bone marrow-derived metastatic neuroblastoma reveals molecular mechanisms of metabolic reprogramming. Sci. Rep. 15, 28519. 10.1038/s41598-025-13626-8 PMC12325718 40764361

[B8] Cui Z. Fu Y. Zhou M. Feng H. Zhang L. Ma S. (2024). Pan-cancer investigation of RFX family and associated genes identifies RFX8 as a therapeutic target in leukemia. Heliyon 10, e35368. 10.1016/j.heliyon.2024.e35368 PMC11336603 39170430

[B9] Dai C. Shen B. Liu S. Li C. Yang S. Wang J. (2025). Pharmacologic inhibition of CSF-1R suppresses intrinsic tumor cell growth in osteosarcoma with CSF-1R overexpression. J. Transl. Med. 23, 900. 10.1186/s12967-025-06920-6 PMC12341233 40797330

[B10] Dimitrov D. Schäfer P. S. L. Farr E. Rodriguez-Mier P. Lobentanzer S. Badia-I-Mompel P. (2024). LIANA+ provides an all-in-one framework for cell-cell communication inference. Nat. Cell Biol. 26, 1613–1622. 10.1038/s41556-024-01469-w PMC11392821 39223377

[B11] Fan L. He Y. Han J. Ybuan P. Guo X. Wang W. (2018). The osteoarthritis-associated gene PAPSS2 promotes differentiation and matrix formation in ATDC5 chondrogenic cells. Exp. Ther. Med. 16, 5190–5200. 10.3892/etm.2018.6843 30546414 PMC6256856

[B12] Feng W. He M. Jiang X. Liu H. Xie T. Qin Z. (2021). Single-Cell RNA sequencing reveals the migration of osteoclasts in giant cell tumor of bone. Front. Oncol. 11, 715552. 10.3389/fonc.2021.715552 PMC8421549 34504794

[B13] Geng Q. Xu J. Cao X. Wang Z. Jiao Y. Diao W. (2024). PPARG-mediated autophagy activation alleviates inflammation in rheumatoid arthritis. J. Autoimmun. 146, 103214. 10.1016/j.jaut.2024.103214 38648706

[B14] Georgess D. Machuca-Gayet I. Blangy A. Jurdic P. (2014). Podosome organization drives osteoclast-mediated bone resorption. Cell Adh Migr. 8, 191–204. 10.4161/cam.27840 24714644 PMC4198343

[B15] Gomez-Mascard A. Van Acker N. Cases G. Mancini A. Galanou S. Frenois F. X. (2024). Intratumoral heterogeneity assessment of the extracellular bone matrix and immune microenvironment in Osteosarcoma using digital imaging to predict therapeutic response. Lab. Invest 104, 102122. 10.1016/j.labinv.2024.102122 39098628

[B16] Hansen M. S. Madsen K. Price M. Sondergaard T. E. Delaisse J.-M. Soe K. (2024). Transcriptional reprogramming during human osteoclast differentiation identifies regulators of osteoclast activity. Bone Res. 12, 5. 10.1038/s41413-023-00312-6 PMC10806178 38263167

[B17] Hirose K. Ishimoto T. Usami Y. Sato S. Oya K. Nakano T. (2020). Overexpression of Fam20C in osteoblast *in vivo* leads to increased cortical bone formation and osteoclastic bone resorption. Bone 138, 115414. 10.1016/j.bone.2020.115414 32416287

[B18] Hu X. Du N. Song Y. Lang K. Tong W. Ye Q. (2026a). Pan-cancer bone metastasis atlas at single-cell resolution identifies a distinct tumor-associated macrophage subset for mediating Denosumab-induced immunosensitization in lung cancer bone metastasis. Int. J. Biol. Sci. 22, 365–386. 10.7150/ijbs.119777 PMC12681936 41362730

[B19] Hu X. Wu H. Zheng B. Liang C. Shi B. Ye Q. (2026b). The single cell transcriptomic landscape of recurrent giant cell tumor of bone following neoadjuvant denosumab therapy. J. Genet. Genomics S1673-8527 (26), 00071–00078. 10.1016/j.jgg.2026.03.001 41786203

[B20] Jiang N. Hu Z. Wang Q. Hao J. Yang R. Jiang J. (2024). Fibroblast growth factor 2 enhances BMSC stemness through ITGA2-dependent PI3K/AKT pathway activation. J. Cell Physiol. 239, e31423. 10.1002/jcp.31423 39188080

[B21] Kamimoto K. Stringa B. Hoffmann C. M. Jindal K. Solnica-Krezel L. Morris S. A. (2023). Dissecting cell identity *via* network inference and *in silico* gene perturbation. Nature 614, 742–751. 10.1038/s41586-022-05688-9 PMC9946838 36755098

[B22] Kawaida R. Ohtsuka T. Okutsu J. Takahashi T. Kadono Y. Oda H. (2003). Dimerization protein 2 (JDP2), a member of the AP-1 family of transcription factor, mediates osteoclast differentiation induced by RANKL. J. Exp. Med. 197, 1029–1035. 10.1084/jem.20021321 12707301 PMC2193879

[B23] Kottmann V. Nienhaus M. Drees P. Gercek E. Ritz U. (2026). From bone homeostasis to skeletal metastasis and osteosarcoma: insights into osteoclast and osteoblast roles in bone remodelling and cancer. Biochim. Biophys. Acta Rev. Cancer 1881, 189551. 10.1016/j.bbcan.2026.189551 41643948

[B24] Kuleshov M. V. Jones M. R. Rouillard A. D. Fernandez N. F. Duan Q. Wang Z. (2016). Enrichr: a comprehensive gene set enrichment analysis web server 2016 update. Nucleic Acids Res. 44, W90–W97. 10.1093/nar/gkw377 27141961 PMC4987924

[B25] Lee K.-D. Dawson P. A. Summers K. M. (2025). Sulfate biology genes are associated with mineralization in mouse and human. JBMR Plus 9, ziaf130. 10.1093/jbmrpl/ziaf130 PMC12448385 40978129

[B26] Li J. Bai Y. Zhang H. Chen T. Shang G. (2024). Single-cell RNA sequencing reveals the communications between tumor microenvironment components and tumor metastasis in osteosarcoma. Front. Immunol. 15, 1445555. 10.3389/fimmu.2024.1445555 PMC11422128 39324133

[B27] Liang X. Qin F. Yuan Z. Wu M. Zhang J. Liu X. (2026). Colorectal cancer-derived osteopontin rewires macrophages into a pro-metastatic M2 state *via* the PI3K/AKT/CSF1-CSF1R axis. Cell Death Discov. 12, 92. 10.1038/s41420-026-02945-y PMC12894849 41639044

[B28] Lin W. Li Y. Qiu C. Zou B. Gong Y. Zhang X. (2025). Mapping the spatial atlas of the human bone tissue integrating spatial and single-cell transcriptomics. Nucleic Acids Res. 53, gkae1298. 10.1093/nar/gkae1298 PMC11736439 39817519

[B29] Lin Q. Luo J. Duan Z. Luo J. Zhang W. Xia Y. (2026). Targeting RANKL-independent osteoclastogenesis overcomes denosumab resistance in models of ER+ breast cancer bone metastasis. J. Clin. Invest 136, e199285. 10.1172/JCI199285 PMC13178652 42138086

[B30] Liu F. Ding Y. Xu Z. Hao X. Pan T. Miles G. (2025a). Single-cell profiling of bone metastasis ecosystems from multiple cancer types reveals convergent and divergent mechanisms of bone colonization. Cell Genom 5, 100888. 10.1016/j.xgen.2025.100888 PMC12278635 40412393

[B31] Liu J. Ren B. He T. Li D. Ding T. Wang Q. (2025b). Engineering Osteosarcoma *in vitro:* from traditional models to biofabricated platforms for precision medicine. ACS Omega 10, 55219–55233. 10.1021/acsomega.5c09120 PMC12658639 41322649

[B32] Liu Y. Luo W. Xu Y. Yao X. Dai L. Feng Q. (2026). Single-Cell sequencing reveals the immunosuppressive trajectory in the tumor microenvironment of human giant cell tumor of bone. Biomed. Res. Int. 2026, 9855803. 10.1155/bmri/9855803 PMC12862500 41635288

[B33] Lopez R. Regier J. Cole M. B. Jordan M. I. Yosef N. (2018). Deep generative modeling for single-cell transcriptomics. Nat. Methods 15, 1053–1058. 10.1038/s41592-018-0229-2 30504886 PMC6289068

[B34] Luecken M. D. Buttner M. Chaichoompu K. Danese A. Interlandi M. Mueller M. F. (2022). Benchmarking atlas-level data integration in single-cell genomics. Nat. Methods 19, 41–50. 10.1038/s41592-021-01336-8 PMC8748196 34949812

[B35] McDonald M. M. Khoo W. H. Ng P. Y. Xiao Y. Zamerli J. Thatcher P. (2021). Osteoclasts recycle *via* osteomorphs during RANKL-stimulated bone resorption. Cell 184, 1330–1347.e13. 10.1016/j.cell.2021.02.002 PMC7938889 33636130

[B36] Moquin-Beaudry G. Marques Da Costa M. E. Khneisser P. Thuilliez C. Audinot B. Zair H. (2026). Spatial transcriptomic atlas of aggressive osteosarcomas reveals shared immune landscape and targetable surface markers. Nat. Commun. 17, 8294. 10.1038/s41467-026-74603-x PMC13470005 42399228

[B37] Patel J. J. Zhu D. Opdebeeck B. D’Haese P. Millán J. L. Bourne L. E. (2018). Inhibition of arterial medial calcification and bone mineralization by extracellular nucleotides: the same functional effect mediated by different cellular mechanisms. J. Cell Physiol. 233, 3230–3243. 10.1002/jcp.26166 28976001 PMC5792173

[B38] Petersen R. B. Buch C. D. Faergemann C. Nymark T. (2025). Bone deformities with hereditary multiple osteochondromas. Dan. Med. J. 72, A11230696. 10.61409/A11230696 40407290

[B39] Seals D. F. Azucena E. F. Pass I. Tesfay L. Gordon R. Woodrow M. (2005). The adaptor protein Tks5/Fish is required for podosome formation and function, and for the protease-driven invasion of cancer cells. Cancer Cell 7, 155–165. 10.1016/j.ccr.2005.01.006 15710328

[B40] Squair J. W. Gautier M. Kathe C. Anderson M. A. James N. D. Hutson T. H. (2021). Confronting false discoveries in single-cell differential expression. Nat. Commun. 12, 5692. 10.1038/s41467-021-25960-2 PMC8479118 34584091

[B41] Su Z. Yeung M. C. F. Han S. Yau R. C. H. Lam Y. L. Ho K. W. Y. (2025). Denosumab enhances antitumor immunity by suppressing SPP1 and boosting cytotoxic T cells. Cancer Immunol. Res. 13, 646–660. 10.1158/2326-6066.CIR-24-1094 40009710

[B42] Sugiaman-Trapman D. Vitezic M. Jouhilahti E.-M. Mathelier A. Lauter G. Misra S. (2018). Characterization of the human RFX transcription factor family by regulatory and target gene analysis. BMC Genomics 19, 181. 10.1186/s12864-018-4564-6 29510665 PMC5838959

[B43] Sun L. Zhang J. Xiahou Z. Zhao Z. Liang Y. (2024). Single-cell RNA sequencing revealed PPARG promoted osteosarcoma progression: based on osteoclast proliferation. Front. Immunol. 15, 1506225. 10.3389/fimmu.2024.1506225 PMC11810940 39936154

[B44] Takayanagi H. (2007). Osteoimmunology: shared mechanisms and crosstalk between the immune and bone systems. Nat. Rev. Immunol. 7, 292–304. 10.1038/nri2062 17380158

[B45] Toda Y. Ogura K. Iwata S. Kobayashi E. Osaki S. Fukushima S. (2026). Identification of TRACP 5b as a local recurrence biomarker in giant cell tumor of bone. Surg. Oncol. 64, 102331. 10.1016/j.suronc.2025.102331 41273883

[B46] UzunM. F. KafadarI. H. CakarB. GolgeliogluF. AydinA. AtahanM. O. (2026). Campanacci stage and cortical destruction predict functional outcomes in giant cell tumor of bone. Ir. J. Med. Sci. 10.1007/s11845-026-04458-0 42154415

[B47] Wang S. Ma F. Wang D. Yu Q. Zheng H. Chen Z. (2026). A pan-cancer single-cell transcriptomic atlas of human bone metastases. Cell Rep. Med. 7, 102583. 10.1016/j.xcrm.2025.102583 PMC12923970 41619722

[B48] Wei H. Shi K. Huang D. Lin H. Wang S. Chen X. (2025). The integrin α2-osteoclast axis: a key driver of bone destruction and therapeutic target in osteosarcoma. J. Transl. Med. 23, 1204. 10.1186/s12967-025-06906-4 PMC12577262 41174632

[B49] Wolf F. A. Angerer P. Theis F. J. (2018). SCANPY: large-scale single-cell gene expression data analysis. Genome Biol. 19, 15. 10.1186/s13059-017-1382-0 29409532 PMC5802054

[B50] Wu X. Deng W. Zhao Q. Xiong J. (2025). Single-cell analysis links DCUN1D5 to immune remodeling and cisplatin resistance in recurrent osteosarcoma. Commun. Biol. 8, 1019. 10.1038/s42003-025-08409-w PMC12238620 40629059

[B51] Yang S. Corbett S. E. Koga Y. Wang Z. Johnson W. E. Yajima M. (2020). Decontamination of ambient RNA in single-cell RNA-seq with DecontX. Genome Biol. 21, 57. 10.1186/s13059-020-1950-6 32138770 PMC7059395

[B52] Yang C. Lai Y. Wang J. Chen Q. Pan Q. Xu C. (2024). Spatial heterogeneity of PD-1/PD-L1 defined Osteosarcoma microenvironments at single-cell spatial resolution. Lab. Invest 104, 102143. 10.1016/j.labinv.2024.102143 39321925

[B53] Yao J. Xie Y. Gao L. Liu G. Xu L. Zhang Y. (2026). Recurrence of giant cell tumor of bone and related risk factors: a retrospective analysis of 340 cases from a single Center. J. Surg. Oncol. 134, 299–306. 10.1002/jso.70295 42226602 PMC13427144

[B54] Zehenter J. I. Kager L. Eder S. K. Taschner-Mandl S. Hinić S. (2026a). New biological insights into osteosarcoma-lessons from single sequencing studies. Cancer Metastasis Rev. 45, 29. 10.1007/s10555-026-0336-z PMC13149624 42091739

[B55] Zehenter J. I. Kager L. Eder S. K. Taschner-Mandl S. Hinić S. (2026b). New biological insights into osteosarcoma-lessons from single cell sequencing studies. Cancer Metastasis Rev. 45, 29. 10.1007/s10555-026-10336-z PMC13149624 42091739

[B56] Zhang W. Bado I. L. Hu J. Wan Y.-W. Wu L. Wang H. (2021). The bone microenvironment invigorates metastatic seeds for further dissemination. Cell 184, 2471–2486.e20. 10.1016/j.cell.2021.03.011 33878291 PMC8087656

[B57] Zhang N. Haizhen Z. Zhang R. Li X. (2024a). Machine learning-based selection of immune cell markers in osteosarcoma: prognostic determination and validation of CLK1 in disease progression. Front. Immunol. 15, 1468875. 10.3389/fimmu.2024.1468875 PMC11685004 39742274

[B58] Zhang W. Wan Z. Qu D. Sun W. Zhang L. Liang Y. (2024b). Profibrogenic macrophage-targeted delivery of mitochondrial protector *via* exosome formula for alleviating pulmonary fibrosis. Bioact. Mater 32, 488–501. 10.1016/j.bioactmat.2023.09.019 PMC10641087 37965241

[B59] Zhang H. Wu S. Luo D. Guo J. (2026a). Computational discovery of novel colony-stimulating factor-1 receptor as a potential therapeutic biomarker osteosarcoma and a novel inhibitor from herbal sources. Oncol. Lett. 31, 263. 10.3892/ol.2026.1618 PMC13145329 42100022

[B60] Zhang H. Wu S. Luo D. Guo J. (2026b). Computational discovery of novel colony-stimulating factor-1 receptor as a potential therapeutic biomarker in osteosarcoma and a novel inhibitor from herbal sources. Oncol. Lett. 31, 263. 10.3892/ol.2026.15618 PMC13145329 42100022

[B61] Zhu G. Chen W. Tang C.-Y. McVicar A. Edwards D. Wang J. (2022). Knockout and Double knockout of Cathepsin K and Mmp9 reveals a novel function of Cathepsin K as a regulator of osteoclast gene expression and bone homeostasis. Int. J. Biol. Sci. 18, 5522–5538. 10.7150/ijbs.72211 36147479 PMC9461675

[B62] Zhu X. Zhou X. Li S. Liu Z. Yu S. Shi H. (2025). PFKFB3 decreases α-ketoglutarate production while partial PFKFB3 knockdown in macrophages ameliorates arthritis in tumor necrosis factor-transgenic mice. Int. Immunopharmacol. 148, 114102. 10.1016/j.intimp.2025.114102 39870011

[B63] Zimmerman K. D. Espeland M. A. Langefeld C. D. (2021). A practical solution to pseudoreplication bias in single-cell studies. Nat. Commun. 12, 738. 10.1038/s41467-021-21038-1 33531494 PMC7854630

[B64] Zuo H. Yang D. Wan Y. (2021a). Fam20C regulates bone resorption and breast cancer bone metastasis through Osteopontin BMP4. Cancer Res. 81, 5242–5254. 10.1158/008-5472.CAN-20-3328 34433585

[B65] Zuo H. Yang D. Wan Y. (2021b). Fam20C regulates bone resorption and breast cancer bone metastasis through Osteopontin and BMP4. Cancer Res. 81, 5242–5254. 10.1158/0008-5472.CAN-20-3328 34433585

