# Supplementary material for: Tumor-associated osteoclasts converge on a matrix-sulfation program across human bone tumors
Source: Front Cell Dev Biol. 2026 Sep 10;14:1914698. doi: 10.3389/fcell.2026.1914698 (PMC13601788; doi:10.3389/fcell.2026.1914698)

Supplementary Figure 3 | Biological-replicate statistics and artifact sensitivity

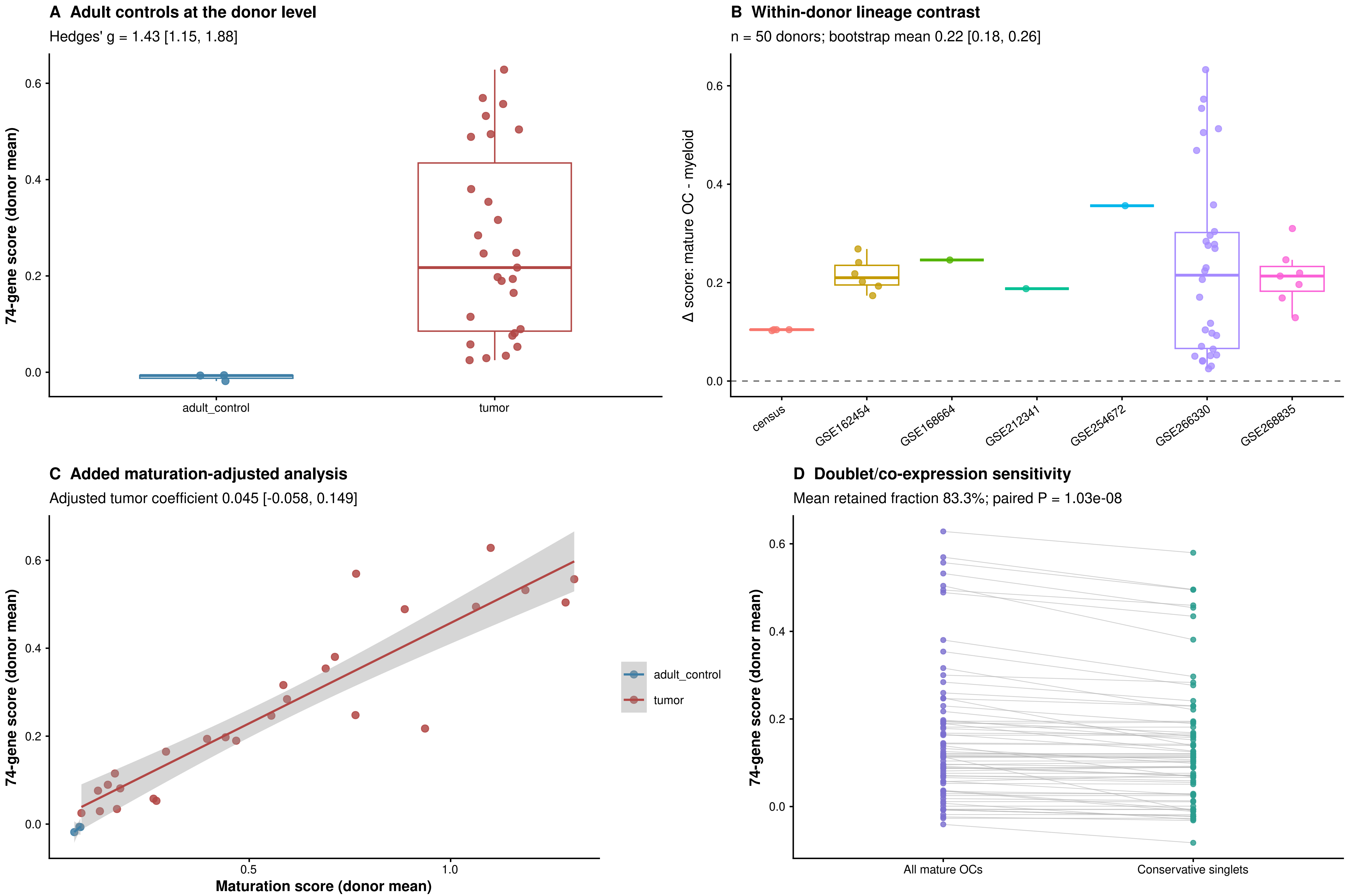

Supplement: Supplementary file 7 [file DataSheet3.pdf]
