# Supplementary material for: Tumor-associated osteoclasts converge on a matrix-sulfation program across human bone tumors
Source: Front Cell Dev Biol. 2026 Sep 10;14:1914698. doi: 10.3389/fcell.2026.1914698 (PMC13601788; doi:10.3389/fcell.2026.1914698)

Supplementary Figure 4 | Independent eight-donor human osteoclast time course

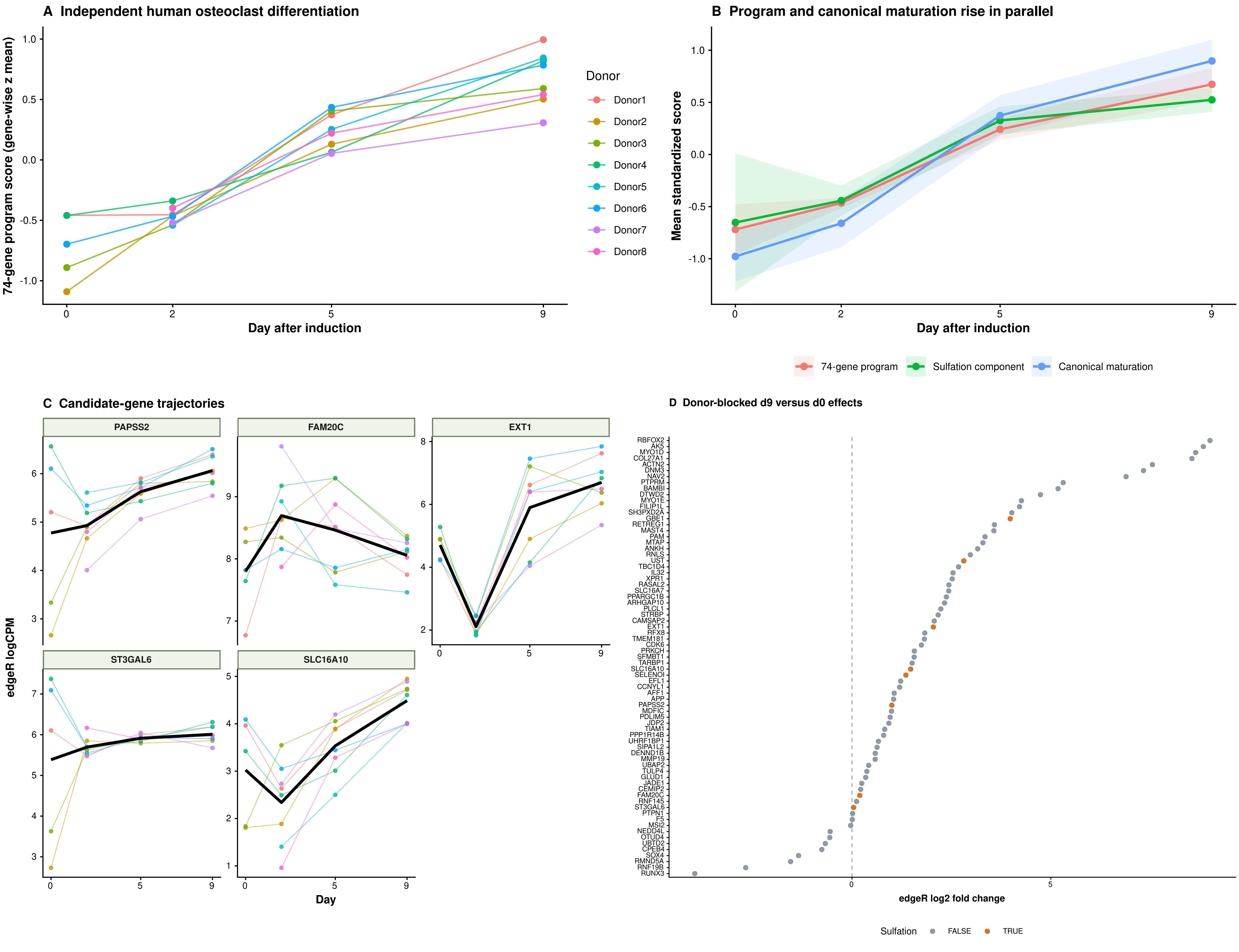

Supplement: Supplementary file 8 [file DataSheet4.pdf]
